# Mobile Monitoring and Embedded Control System for Factory Environment

**DOI:** 10.3390/s131217379

**Published:** 2013-12-17

**Authors:** Kuang-Yow Lian, Sung-Jung Hsiao, Wen-Tsai Sung

**Affiliations:** 1 Department of Electrical Engineering, National Taipei University of Technology, No.1, Sec. 3, Zhongxiao E. Rd., Taipei 10608, Taiwan; E-Mails: kylian@mail.ntut.edu.tw (K.-Y.L.); t100319007@ntut.edu.tw (S.-J.H.); 2 Department of Electrical Engineering, National Chin-Yi University of Technology, No.57, Sec. 2, Zhongshan Rd., Taiping Dist., Taichung 41170, Taiwan

**Keywords:** ZigBee, sensor, Arduino, Wi-Fi, ARM, TCP/IP, FFT, NFC

## Abstract

This paper proposes a real-time method to carry out the monitoring of factory zone temperatures, humidity and air quality using smart phones. At the same time, the system detects possible flames, and analyzes and monitors electrical load. The monitoring also includes detecting the vibrations of operating machinery in the factory area. The research proposes using ZigBee and Wi-Fi protocol intelligent monitoring system integration within the entire plant framework. The sensors on the factory site deliver messages and real-time sensing data to an integrated embedded systems via the ZigBee protocol. The integrated embedded system is built by the open-source 32-bit ARM (Advanced RISC Machine) core Arduino Due module, where the network control codes are built in for the ARM chipset integrated controller. The intelligent integrated controller is able to instantly provide numerical analysis results according to the received data from the ZigBee sensors. The Android APP and web-based platform are used to show measurement results. The built-up system will transfer these results to a specified cloud device using the TCP/IP protocol. Finally, the Fast Fourier Transform (FFT) approach is used to analyze the power loads in the factory zones. Moreover, Near Field Communication (NFC) technology is used to carry out the actual electricity load experiments using smart phones.

## 1. Introduction

This work proposes an intelligent monitoring system suitable for factory area management and control [1,2]. Numerous industrial and work place hazards necessitate a system that monitors the conditions of a factory area. A factory health monitoring system was created to improve industrial processes by reducing the handling of dangerous things time required [3]. By using their smart phones, managers can instantly monitor a factory area. This is a very innovative approach to safeguard a workplace. The proposed system is based on the Arduino web server architecture [4].

This paper presents three main monitoring systems for the measurement of temperature and humidity analog signal detection and digital signal control [5]. These systems are built with an Arduino DUE module. The Arduino DUE is a microcontroller board based on the Atmel SAM3X8E ARM Cortex-M3 CPU. It is the first Arduino board based on a 32-bit ARM core microcontroller. It has 54 digital input/output pins (of which 12 can be used as PWM outputs), 12 analog inputs, four UARTs (hardware serial ports), a 84 MHz clock, an USB OTG capable connection, two DAC (Digital to Analog Converter), two TWI (Two-Wire Interface), a power jack, an SPI header, a JTAG (Joint Test Action Group) header, a reset button and an erase button.

The network connection in this system uses the Arduino Wi-Fi Shield to connect to the base station [6]. The Arduino Wi-Fi Shield allows an Arduino board to connect to the Internet using the 802.11 wireless (Wi-Fi) specification. It is based on the HDG104 Wireless LAN 802.11 b/g System in-Package. An Atmega 32UC3 provides a network (IP) stack capable of both TCP and UDP.

**Figure 1.**
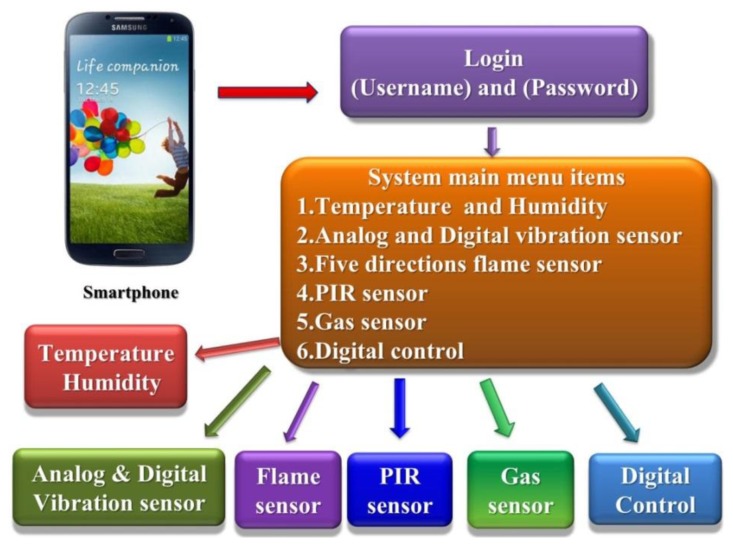
Arduino-based web server system.

Temperature and humidity measurements constitute the first part of this system. The analog signal measurements, including vibration sensors, air quality sensors and luminance sensors, are the second part. The third part is the digital control functions, including the motor operation using a relay circuit control to turn on the lights and air conditioning. Figure 1 shows the proposed system architecture diagram [7]. The main proposed system options include six features: the temperature and humidity sensors, analog and digital vibration sensors, five direction flame sensors, PIR sensor, gas sensor and digital control. Each sensor will send measured data to the integrated controller via ZigBee nodes. The operator can use the mobile device to obtain messages from the remote sensors immediately. If required, the operator can also use a mobile device to turn any remote electronic devices on or off.

**Figure 2.**
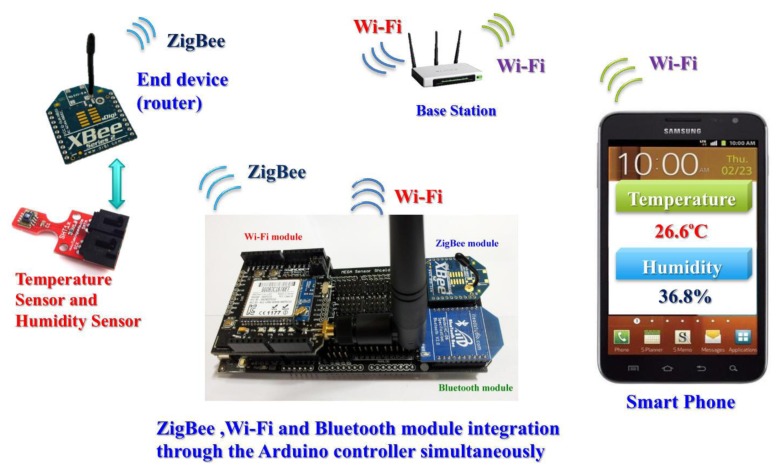
Integrated controller connects devices and sensors by the different protocols.

The monitoring system uses the Wi-Fi protocol to communicate between the smartphone and the Arduino controller. The communication between the sensors and Arduino controller is facilitated by the ZigBee protocol. Figure 2 shows how the temperature data is being sent from the temperature sensor to the smartphone via different protocol platforms. The aforementioned protocols are integrated into the embedded controller by appropriate coding. Sensors and the integrated controller use the ZigBee protocol to communicate with each other in Figure 2. Smart phones and the integrated controller employ the Wi-Fi protocol to transmit information.

Figure 2 shows the integrated controller connecting devices and sensors using different protocols. Wireless sensor networks can ensure reliability and provide adequate security in the factory and industrial domain [8].

## 2. Systems Analysis and Description

**Figure 3.**
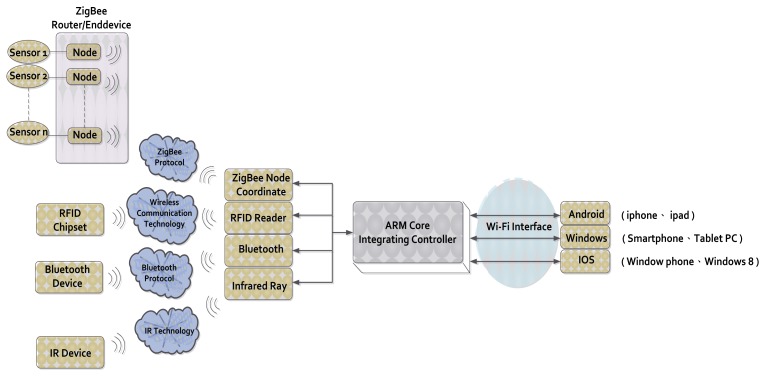
Digital signal convergence technology architecture.

ZigBee and Wi-Fi integrated applications have been intensely discussed [9–11]. This study employs an innovative technology that combines digital signals. Our monitoring system carries out the integration within an Arduino ARM controller. This paper presents real-time ZigBee signals sent to smartphones via the Wi-Fi protocol using our integrated digital controller [12]. Our study presents a controller used to integrate different protocols, therefore, the proposed controller is called integrated controller. Our integrated controller is achieved by writing suitable network programming codes. The integrated controller can integrate various protocols including ZigBee, RFID, Bluetooth, infrared and Wi-Fi. Figure 3 shows our digital signal convergence technology architecture. Our research proposes an innovative approach that integrates different communication protocols using our embedded system. System integration provides the advantages of different protocols including the following features:

- The ZigBee advantages include low power consumption, low cost, high safety, high reliability, and compatibility with each other [9].
- The biggest advantage of RFID is identity confirmation [13].
- Bluetooth and infrared are simple and convenient to utilize. Bluetooth pairing procedures must be completed before they can work in the wireless communication [14].
- The best benefits of Wi-Fi are the coverage area. At present almost all mobile devices support the Wi-Fi protocol.

Our research overcomes the difficulties involved with the requirement for numerous logins. The mobile device does not have any restrictions. This is another innovative technology [15].

## 3. The Introduction and Employ of ZigBee Modules

**Figure 4.**
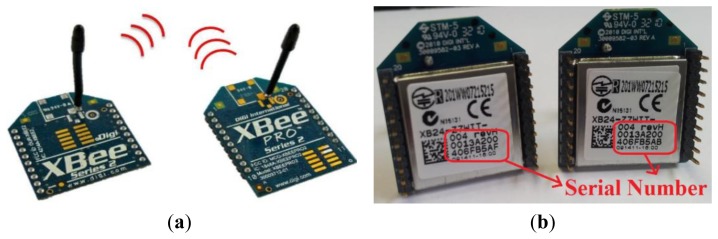
ZigBee modules. (**a**) Antenna on top; (**b**) Network hardware ID on the chip.

Figure 4 presents two ZigBee modules that are integrated in our system. There is a ZigBee network hardware ID on the chip.

Other ZigBee nodes are connected according to this ID. This study constructed an interactive monitoring system using Digi's XBee chip module to create ZigBee nodes [16]. The network layer below ZigBee that supports its advanced features is known as IEEE 802.15.4 [17]. ZigBee is a set of layers built on top of 802.15.4. These layers add three important things:

1. Coordinator: ZigBee networks always have a single coordinator device. Remember that each network must be formed by a coordinator and there will never be more than one coordinator in the network.
2. Router: A router is a full-featured ZigBee node.
3. End device: End devices always need a router or the coordinator as their parent device.

**Figure 5.**
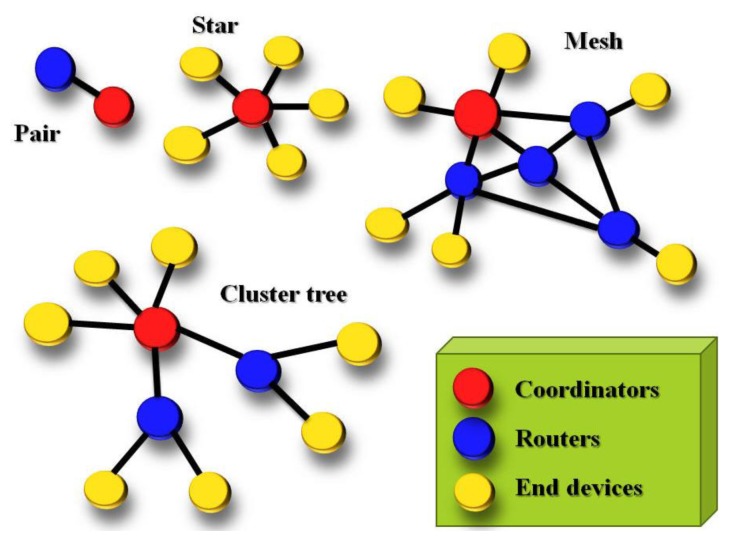
ZigBee network topology.

Figure 5 presents the ZigBee network topology.

**Figure 6.**
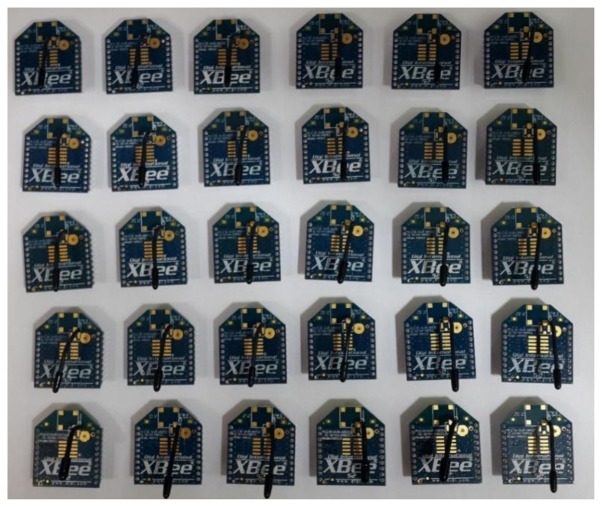
These ZigBee modules are employed to build our monitoring system.

**Figure 7.**
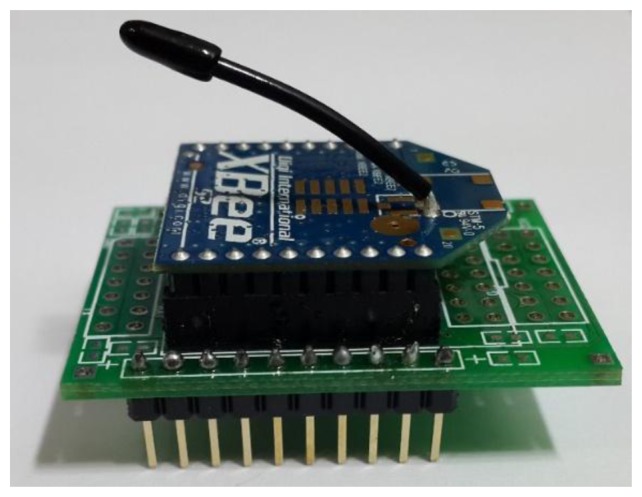
The ZigBee chip module is inserted in the adapter seat.

**Figure 8.**
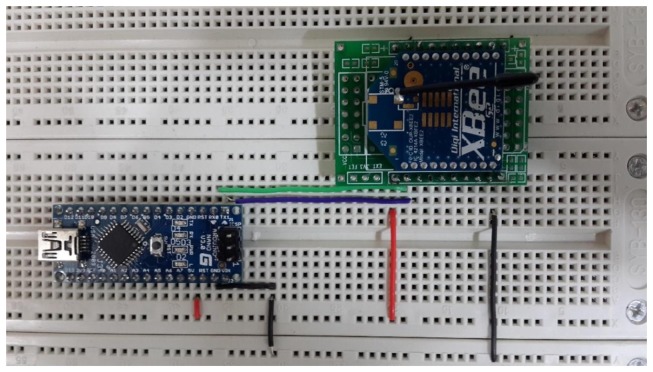
Connecting ZigBee node and Arduino NANO.

**Figure 9.**
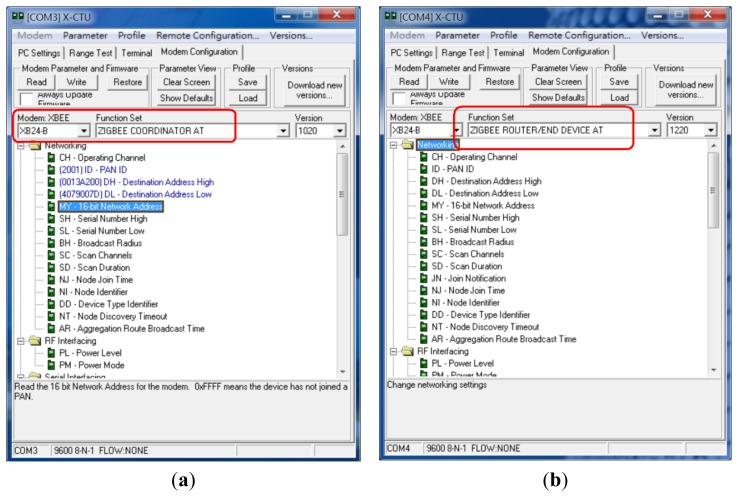
Initializing the ZigBee module network functions. (**a**) Setting a coordinator; (**b**) Setting a router or end device.

The following article presents the ZigBee module assembly and role setting details:

Step 1: First, this research employs many ZigBee modules which will be used to build the proposed system. Figure 6 presents these ZigBee modules.
Step 2: Next, this research will install a ZigBee module adapter seat. The breadboard hole distances and ZigBee chip pin distances are not the same, so this process will construct a homemade adapter seat.
Step 3: This paper uses a soldering iron and solder to weld this adapter seat.
Step 4: The ZigBee chip module is inserted in the adapter seat as shown in Figure 7.
Step 5: These end-sensors combined ZigBee modules with Arduino NANO boards. As shown in Figure 8 the ZigBee module TX pin is connected to the Arduino NANO RX pin. In the same case the RX pin of the ZigBee module is connected to the Arduino NANO TX pin.
Step 6: Setting these ZigBee modules for networking role in our monitoring system, please refer to Figure 9 setting screen.

## 4. Sensors Introduction

This section describes the main sensors used in our system. These sensors include temperature and humidity sensors, analog piezo-disk vibration sensor, digital and analog vibration sensor, five direction flame sensors, passive infrared sensor and gas sensor [18].

### 4.1. Temperature and Humidity

**Figure 10.**
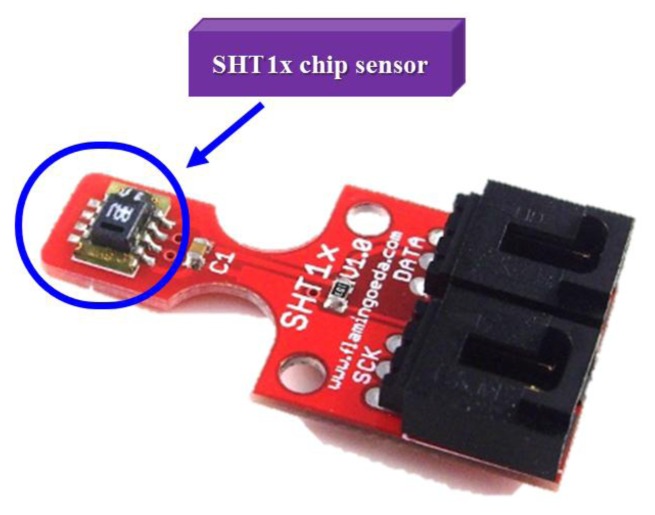
A SHT1x digital temperature and humidity sensor.

**Figure 11.**
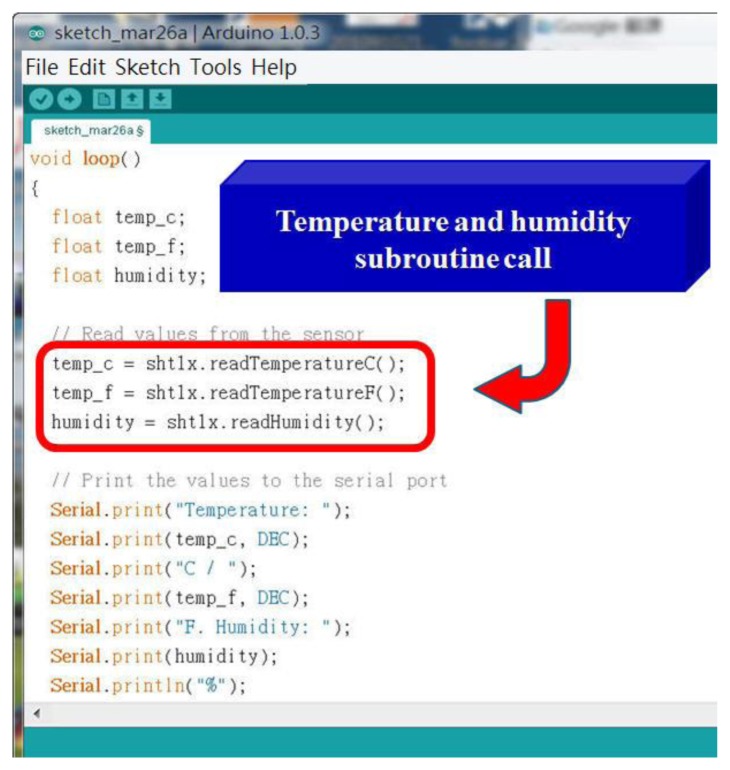
Part of the code for the measurement of temperature and humidity.

**Figure 12.**
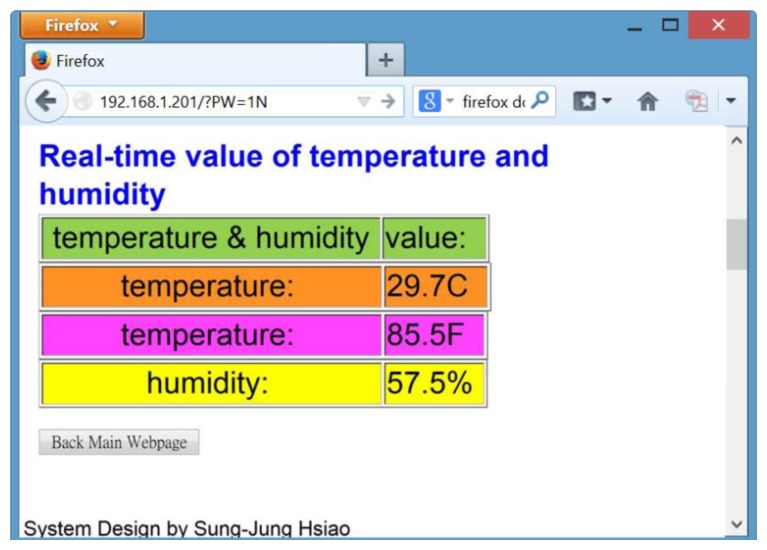
Using the browser to view real-time temperature and humidity data in the integrated controller.

Figure 10 shows the high accuracy digital temperature and humidity sensors. This sensor uses a SHT1x sensor. The SHT1x is individually calibrated in a precision humidity chamber. The calibration coefficients are programmed into an OTP memory on the chip. These coefficients are used to internally calibrate the signals from the sensors. The 2-wire serial interface and internal voltage regulation allows for easy and fast system integration [19]. The tiny size and low power consumption makes SHT1x the ultimate choice for even the most demanding applications. This research uses the C++ language to write temperature and humidity subroutines so that the main program calls them, and the hardware chip measured value is read out. Figure 11 shows that the main program calls the temperature and humidity subroutine. Next this system will write real-time data for temperature and humidity into the HTML format and put this coded information into the embedded system Web Server. Figure 12 shows how the remote real-time temperature and humidity information is presented on the browser. Figure 12 shows the remote temperature and humidity sensor data content to be coded into our integrated controller. Our research draws a line graph showing the temperature and humidity data acquired in the past ten minutes.

**Figure 13.**
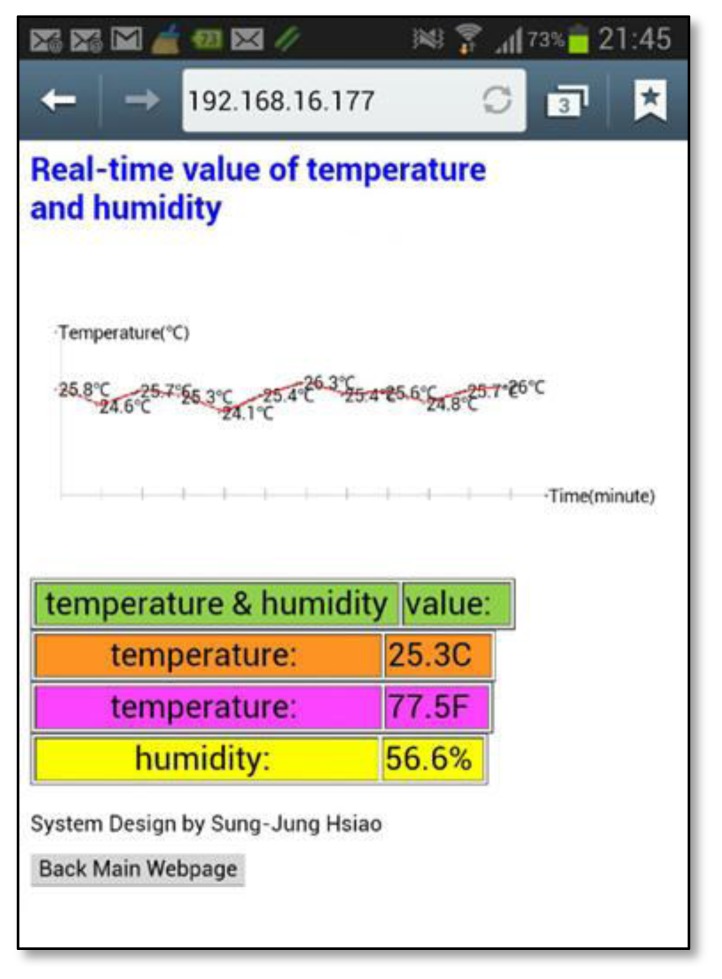
Utilizing a mobile phone to observe the temperature trend in the past 10 min.

This paper used JavaScript technology to draw the historical line graph shown in Figure 13. In addition to using a computer browser, the operator can use any mobile device to link to the temperature and humidity information.

**Figure 14.**
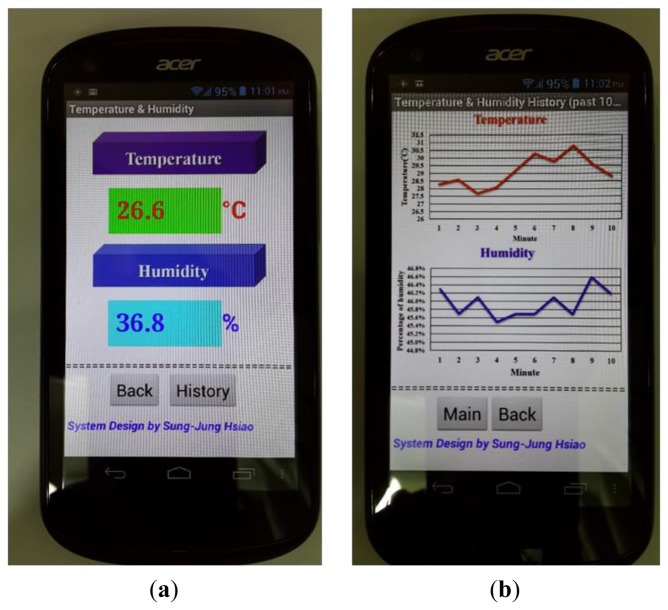
Employing android app methods to link temperature and humidity real-time information. (**a**) Temperature and humidity; (**b**) past 10 min history data.

This study also wrote smart phone APP programing to increase control operational efficiency. The APP approach can be applied to the proposed system by using a smart phone. Figure 14 shows the Android platform used to write real-time temperature and humidity display and drawing APP program. According to our research, the method makes use of an APP program that is the direct link into the Web-Base for better efficiency. On the other hand, the use of APP programs is also more in line with the habits of people's operating mode on smartphones.

### 4.2. Vibration Sensor

There are many types of vibration sensors [20]. The measured signal values can be classified into analog signals and digital signals.

#### 4.2.1. Analog Piezo-Disk Vibration Sensor

**Figure 15.**
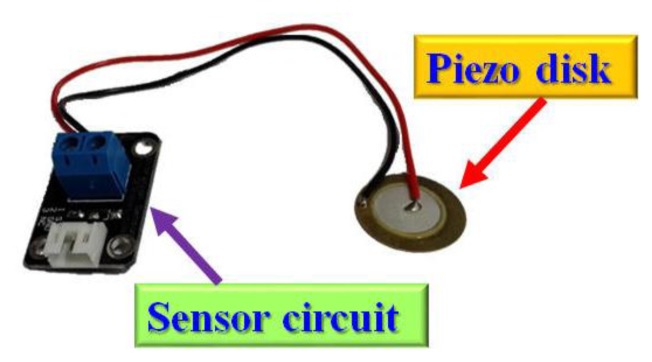
Analog piezo-disk vibration sensor.

The vibration sensor buffers a piezoelectric transducer that responds to strain changes by generating a measurable output voltage change which is proportional with the strength of vibration [21]. Figure 15 shows an analog piezo-disk vibration sensor.

**Figure 16.**
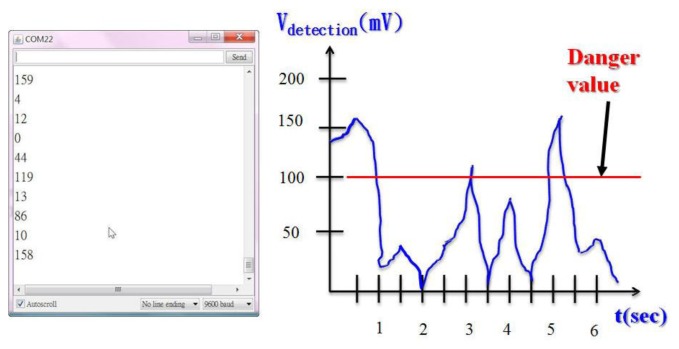
Actual measurement of the vibration signal.

Our system reads the vibration sensing signal from the COM port in the actual situation. The operator is able to obtain the vibration values graphed according to time frequency. This system can arbitrarily adjust the measurement point time period. Figure 16 presents an actual measurement of the vibration signal. In Figure 16 the system programed measurement time point is 0.5 s. This vibration sensor detects a voltage threshold of 100 mV as safe. If the range is between 80 mV and 100 mV a warning value is given. If the value exceeds 100 mV an alert will immediately sound.

**Figure 17.**
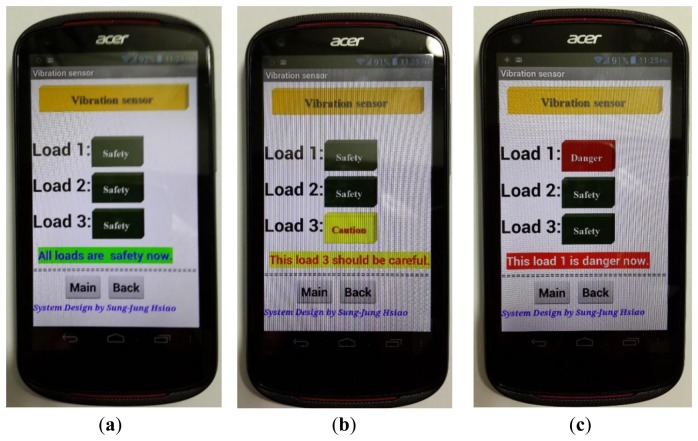
Vibration sensor and voltage detection range. (**a**) V_detection_ < 80 mV; (**b**) 80mV ≤ V_detection_ ≤ 100 mV; (**c**) V_detection_ > 100 mV.

Figure 17 shows that the vibration sensor detects the voltage range corresponding to the smart phone APP interfaces.

Table 1 lists the vibration sensors to detect the voltage range and actual situation.

#### 4.2.2. Digital and Analog Vibration Sensor

In addition to using analog vibration sensors, this research also uses a vibration sensor with digital output as a secondary detection system. The vibration sensor's main chips are LM393 and 801S vibration probes. This vibration sensor works in a DC 3∼5 V voltage range. The sensor features include the following:

(1) Includes a signal output indications
(2) Has analog and TTL level signal output
(3) Output valid signal is high and the light turns off
(4) Adjustable sensitivity (fine-tuning)
(5) Vibration detection range and non-directional
(6) With mounting hole, firmware installation is flexible and convenient

**Figure 18.**
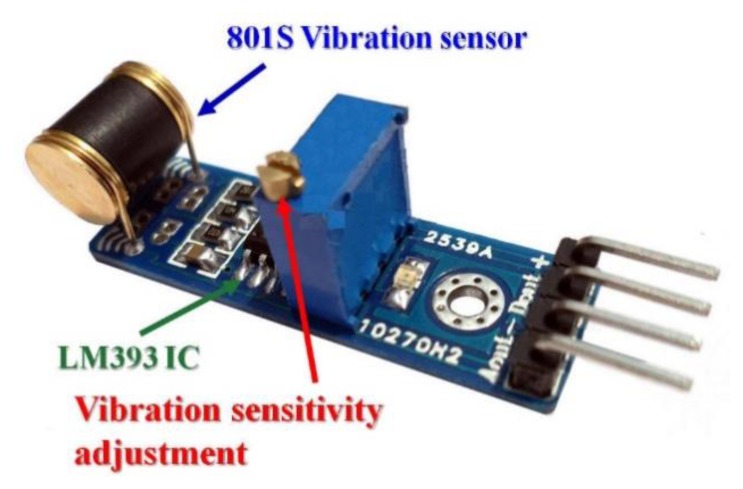
Digital and analog vibration sensor.

Both digital and analog output signals of the vibration sensor are shown in Figure 18.

The vibration sensor has a digital and analog signal output. This work will adjust its analog output sensitivity, the same as the piezo-disk vibration sensor.

### 4.3. Five Directions Flame Sensor

**Figure 19.**
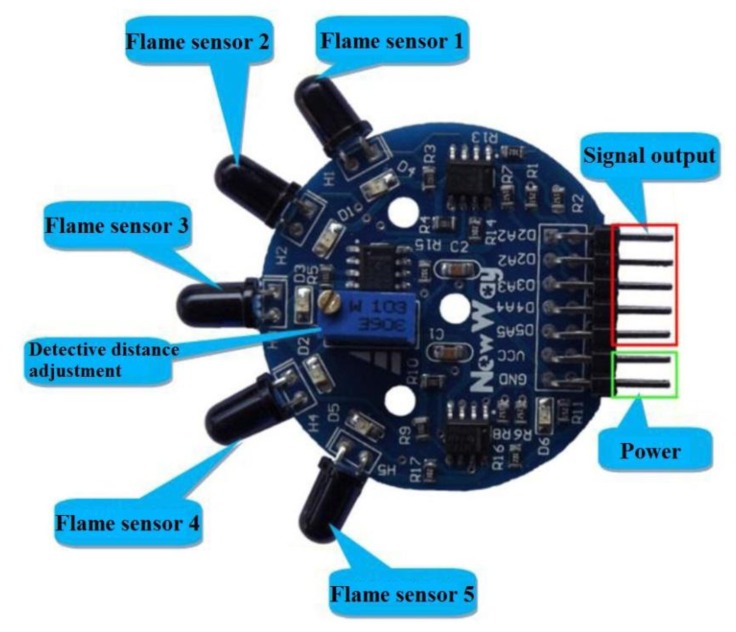
Five directions flame sensor.

The flame sensor can be used to detect fire or other wavelengths at 700 nm–1,100 nm light. The flame sensor probe angle is 120°. There are three M3 mounting holes on the flame sensor module can stabilize the hardware module. The operating temperature range of the flame sensor is −25 °C to 85 °C and in the course of the flame detection it should be noted that the probe distance from the flame should not be too close in order to avoid damage [22]. Figure 19 presents the five direction flame sensor.

**Figure 20.**
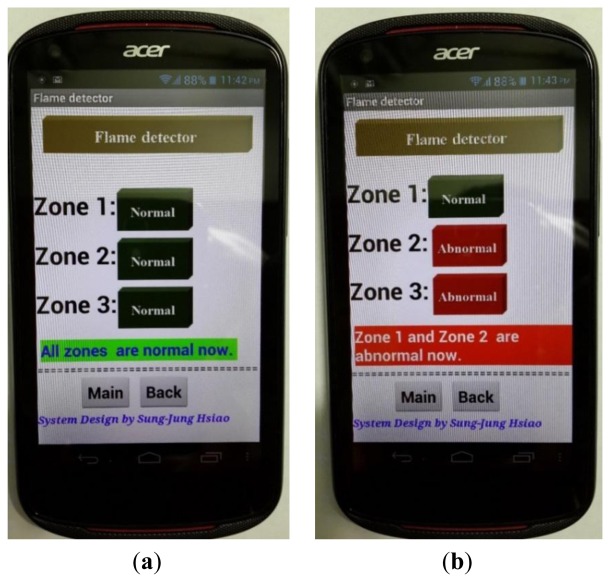
Real-time monitoring each zones situation by these flame sensors. (**a**) Normal situation; (**b**) Abnormal situation.

The Android APP phone detection program was written in the same way. When the flame detector senses an abnormal signal the smartphone monitor will generate an alert screen in addition to the normal factory alarm (refer to Figure 20 for the APP program screen).

### 4.4. PIR (Passive Infrared) Sensor

**Figure 21.**
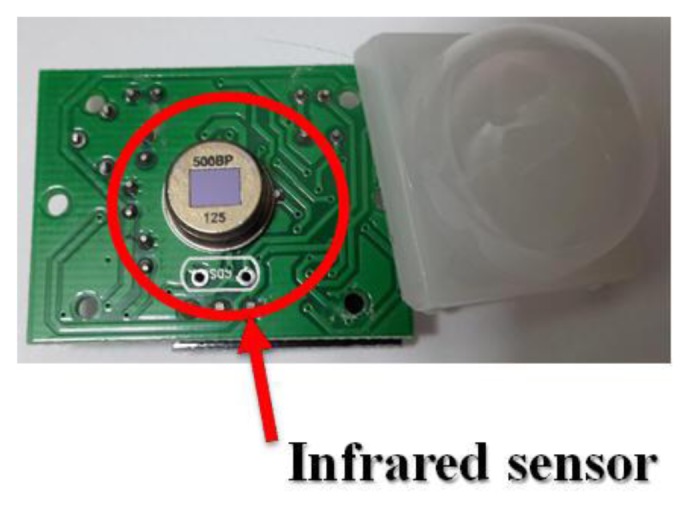
Main infrared sensor component.

There are some areas within a plant that no one can shut down because they are dangerous, especially machines during operation. For this reason a Passive Infrared sensor (PIR) is particularly important [23,24]. When a person or animal approaches the machine a PIR sensor will produce an output signal and respond immediately to anomalies. The distance and sensitivity can be adjusted. Figure 21 presents the main infrared sensor component. The infrared detection sensor is located just above the entire sensor module.

**Figure 22.**
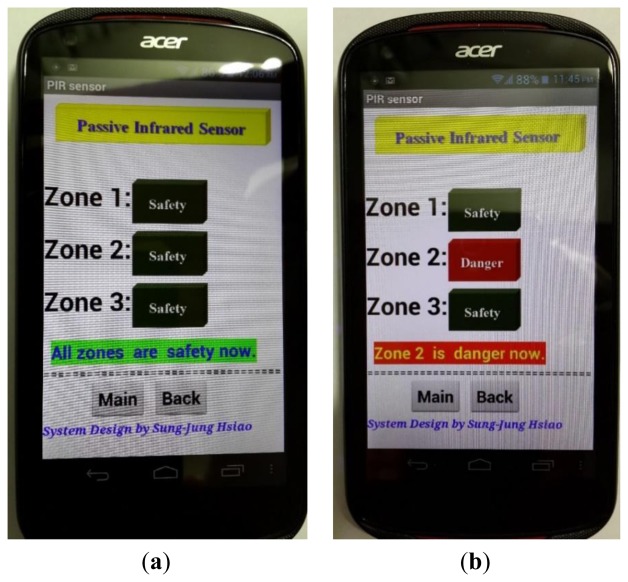
PIR different situation shows on the smartphone. (**a**) Safety situation; (**b**) Showing “Zone 2 is danger now”.

**Figure 23.**
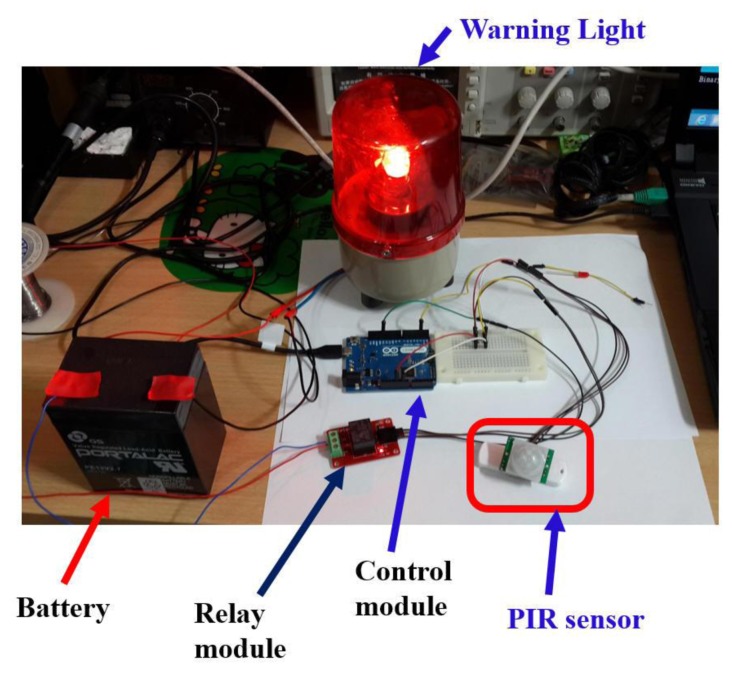
Personnel near the PIR sensor, the warning light will turn on.

This system uses the Android APP program to show whether factory workers are close to the danger zone (refer to Figure 22 for the display). Figure 22a shows that all zones are safe. Figure 22b shows that Zone 2 is in danger. Figure 23 shows when a person is close to the PIR sensor, which activates a warning light and alarm.

### 4.5. Gas Sensor

**Figure 24.**
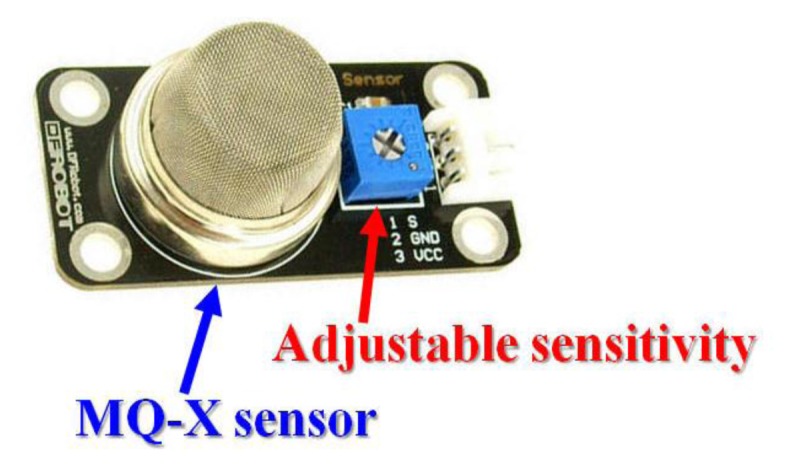
Analog gas quality sensor.

This analog gas sensor detects gas leaks in consumer and industrial appliances [25]. This sensor is suitable for detecting LPG, butane, propane, methane, alcohol and hydrogen gases. The potentiometer sensitivity can be easily adjusted. It has high sensitivity with fast response time. This sensor will return an analog value that represents the intensity of the detected gas. Figure 24 presents an analog gas quality sensor.

**Figure 25.**
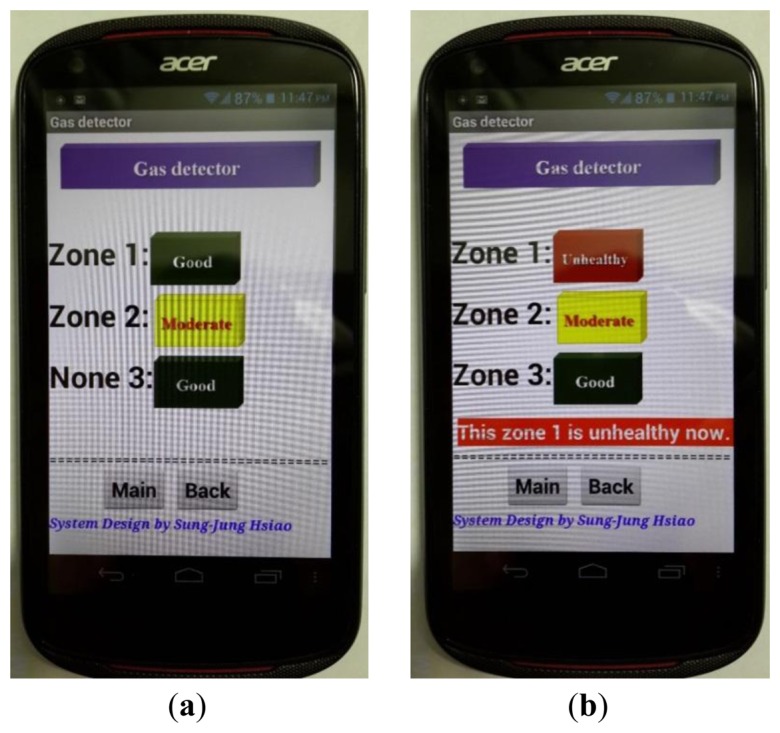
Gas sensor measurement result is displayed in the smart phone. (**a**) Gas sensor detecting moderate air quality; (**b**) Unhealthy air quality display.

Gas sensors can also detect if the air quality is good, moderate or unhealthy. Figure 25 shows the sensor detecting gas quality.

## 5. Power Measurement Module

The Power Measurement Module is a series of small PCB boards that control various power measurement needs. This module obtains complete and accurate power information using a simple communication design [26]. The Power Measurement Module features include the following:

- Voltage (V), current (A), real power (W), apparent power (VA), frequency (Hz), power factor (PF) and the two-way total energy (kWh) measurement and communication transmission function.
- Electrical power measurement from 0.01 W–15 kW; cumulative energy measurement from 0.001 kWh–99,999.999 kWh, very small to high power (W) or energy (kWh) can be measured.
- Power consumption is less than 0.1 W (5 V, 20 mA); current detection circuit power consumption is less than 0.5 W (at rated current)

**Figure 27.**
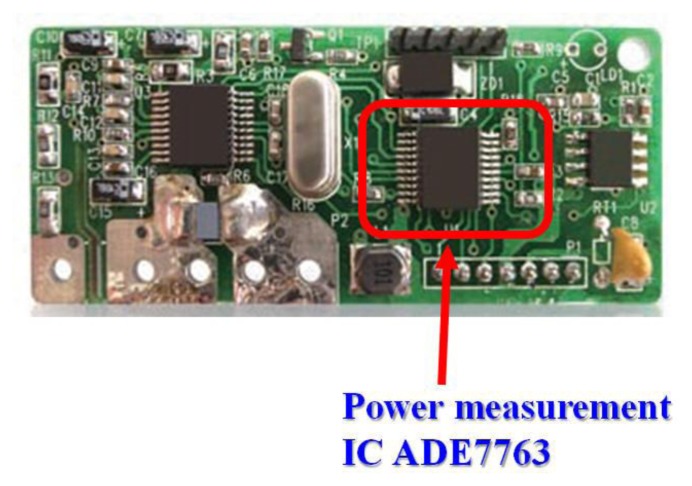
Power Measurement Module.

**Figure 28.**
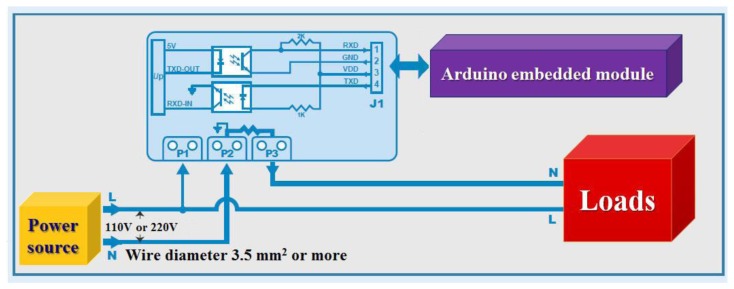
Actual connection with the diagram of each module.

This module is designed to use the ADE7763 power measurement IC with a wide range of voltages and currents. The full range of power measurement is 90–260 V. Figure 27 presents the Power Measurement Module ADE77763 IC position in the PCB figure. The Power Measurement Module connects to each module, please refer to Figure 28.

This system employs the Fast Fourier Transform (FFT) approach to power load analysis. For FFT-related information, please refer to the Appendix below.

## 6. Near Field Communication (NFC)

Near Field Communication (NFC) is a short-range high frequency wireless communication technology that allows electronic devices to execute non-contact-point data transmissions over some 10 cm (9.906 cm), to exchange information. This technology was developed using contactless radio frequency identification (RFID) jointly developed by Philips, Nokia and Sony, based on RFID and interconnection technologies [13]. NFC is a short-range radio frequency technology, in the 13.56 MHz frequency of execution within a distance of 20 cm. Its transmission rate includes 106 kbit/s, 212 kbit/s and 424 kbit/s. Near Field Communication has now passed into the ISO/IEC IS 18092 international standard, EMCA-340 standard and ETSI TS 102 190 standard. NFC uses active and passive read modes [27].

**Figure 29.**
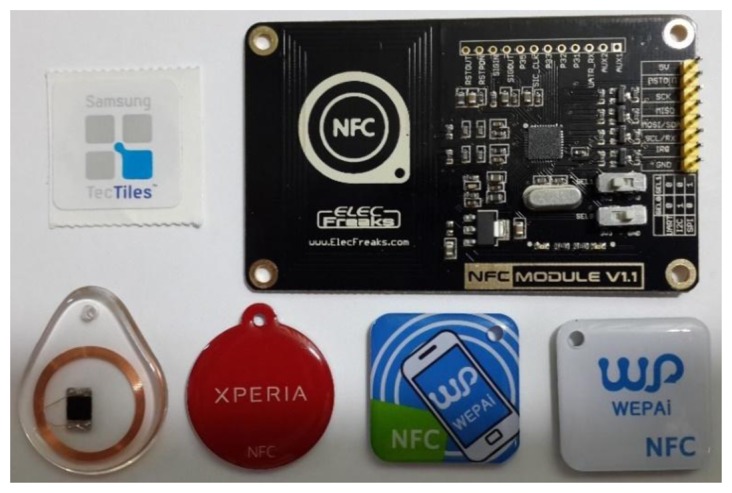
NFC hardware module and various types of tags.

**Figure 30.**
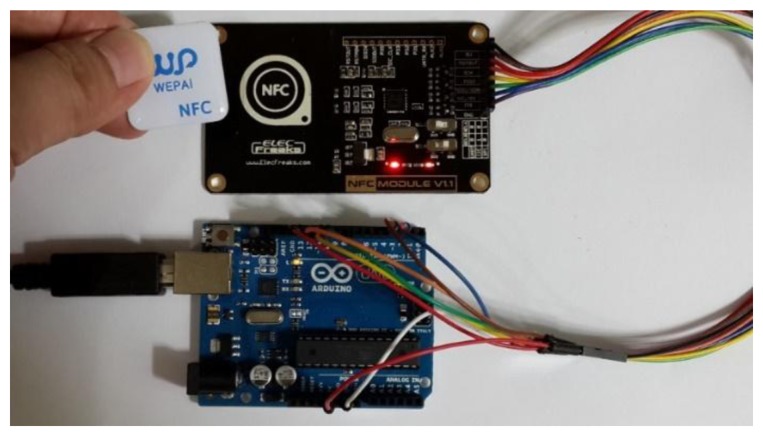
Relevant data or configuration written to a tag using NFC hardware module.

**Figure 31.**
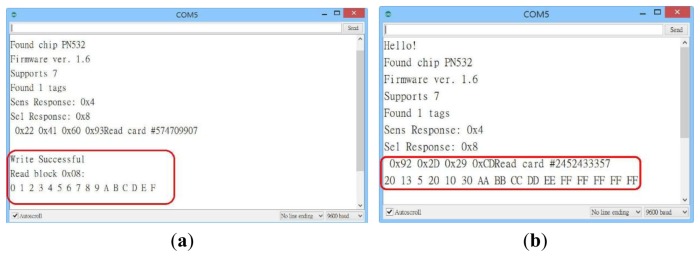
Writing different data to a NFC tag. (**a**) Writing 16 hex data to NFC tag; (**b**) Writing 2013/05/20 data to NFC tag.

This study uses the NFC hardware module to read and write tags [28–30]. Figure 29 presents the NFC hardware module and various types of tags. NFC tags do not all have the same capacity, which ranges from 64 Bytes to 1 kBytes. Our research uses the NFC module to write the configuration to the tag. Relevant data or configuration writes to the tag are shown in Figure 30. According to the NFC tag memory distribution, our study writes the data to the specified storage block. Figure 31 show NFC tags with different written data. Figure 31a presents writing 16 hex data to a NFC tag. Figure 31b shows writing date data to a NFC tag.

**Figure 32.**
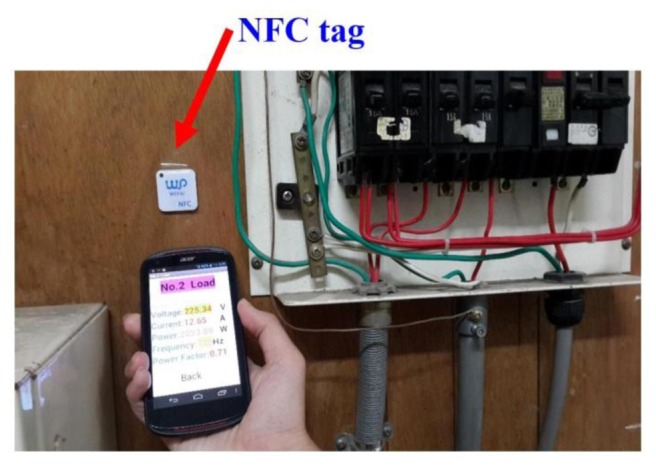
Actually the case of the measured Load 2.

**Figure 33.**
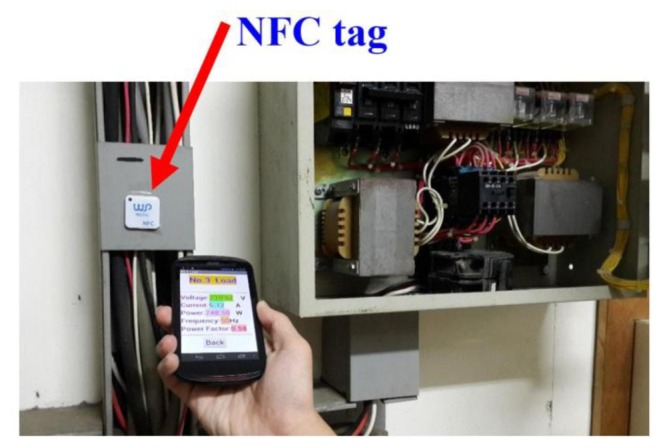
Actually the case of the measured Load 3.

This paper writes startup code to NFC tags. The startup code will allow smartphones to launch a specified program. When a smart phone is close to NFC tags loaded program will be turned on immediately. NFC tags cannot be attached to a metal surface. The benefit of using NFC technology is that it can instantly allow you to view system status. Figures 32 and 33 show NFC tag attached to the Load 2, Load 3 power source side. When this operator brings his smart phone near an NFC tag, the phone will activate the corresponding APP program.

## 7. Overcoming Problems

When this system developed interactive monitoring system many difficulties were encountered. This research had to overcome these difficulties. Here, this work describes two very important challenges and how those challenges were overcome.

### 7.1. Communication Problems and Solutions

**Figure 34.**
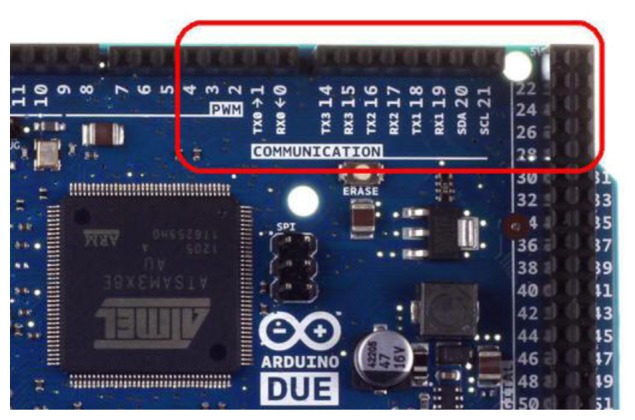
Arduino DUE communication pins with multiple typed pairs.

**Figure 35.**
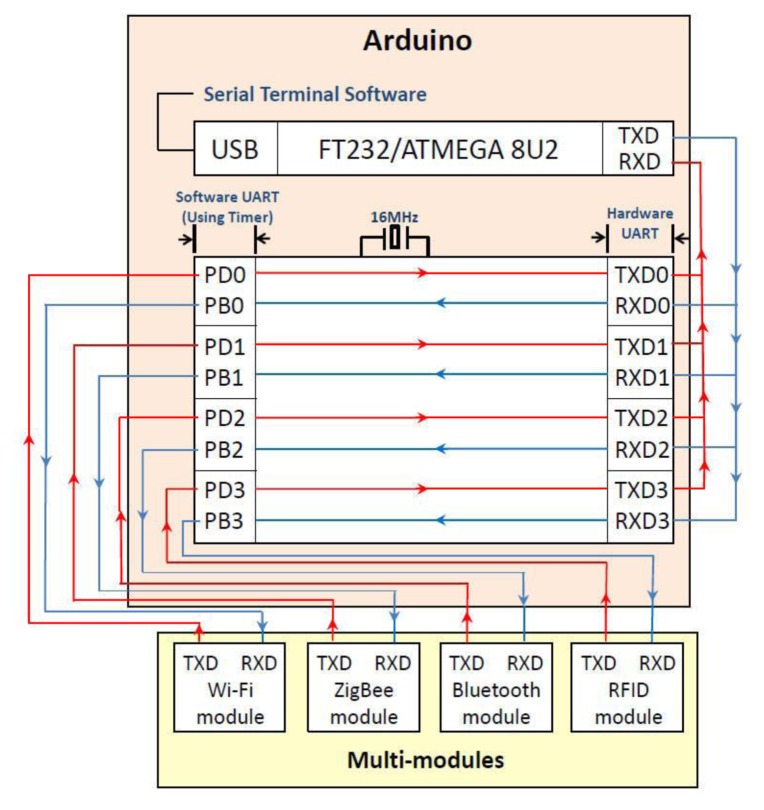
Arduino, Wi-Fi module, ZigBee module, Bluetooth module and RFID module data streams.

This system needs to integrate a variety of digital signals, however, hardware specifications are often not as successful as expected. This paper utilizes the Arduino DUE module to solve the digital signal convergence problem. The Arduino DUE communicates using four pairs of pins, TX0–TX3 and RX0–RX3, as shown in Figure 34. Figure 35 presents the Arduino, Wi-Fi module, ZigBee module, Bluetooth module and RFID module data streams. This study employs multiple pins for communication as the perfect solution to the digital signal convergence problem. When digital signal convergence is resolved using hardware the integrated functions can be completed using the software approach.

### 7.2. Utilizing XML Data Format

**Figure 36.**
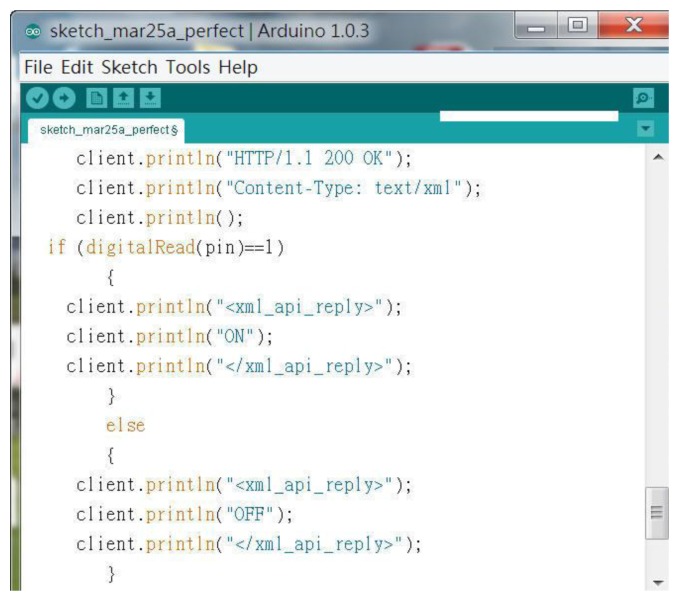
An XML-formatted Web page in C++ codes.

The early Android system could use web information directly, however, with Android 3.0 the page must now use a more rigorous data stream in XML format to make the APP link. Figure 36 is an XML-formatted Web page, but it used the Arduino editor to write C++ codes.

**Figure 37.**
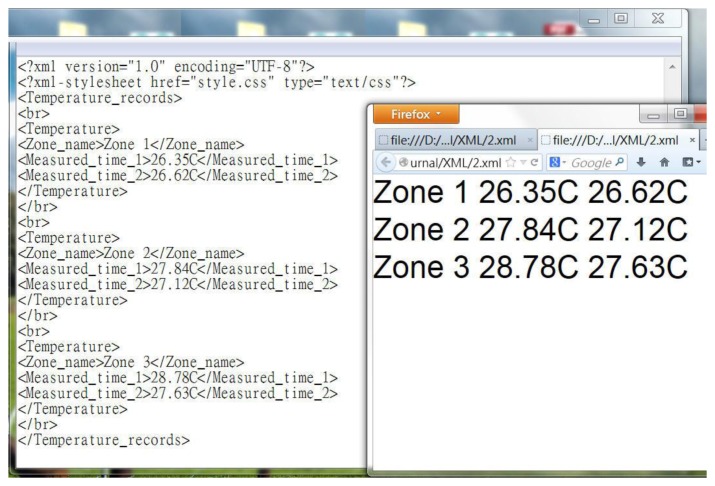
Data properties for easy access.

XML format allows users to know the data properties. Known data properties make this smart phone application easy to access, as shown in Figure 37.

## 8. Implementing the Interactive Monitoring Web Server System

This research employs C++ programming language for our web server. When the remote sensors transfer data to our integrated controller the integrated controller will immediately render the data as a web page. As a way to use web-based Arduino, although let the system runs very smoothly, it only limited data passes in text form. This paper must write a smart phone application to use the drawing function. Therefore, our monitoring system into a first phase stage of development using the web-based Arduino, the second phase of the program is to write a mobile APP.

### 8.1. Employing Arduino Based Web Server System

**Figure 38.**
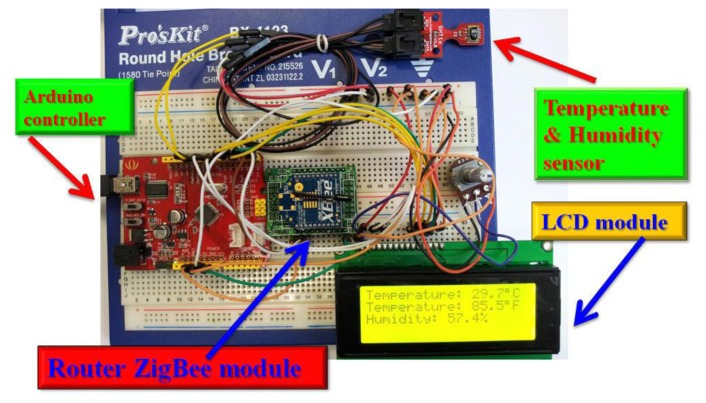
Introduction to the transmitter side of our ZigBee network.

**Figure 39.**
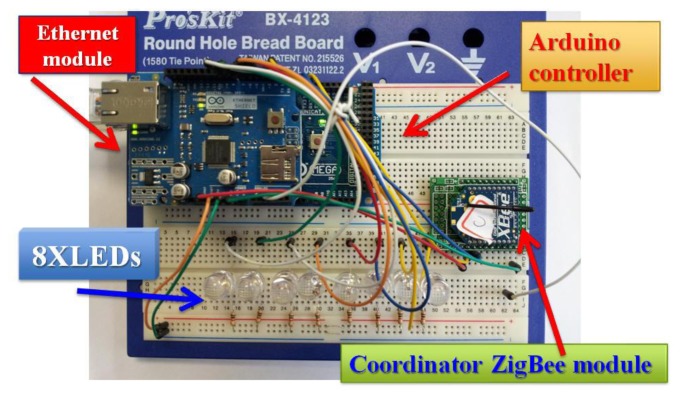
Introduction to the receiver side of our ZigBee network.

This system will use the Wi-Fi Shield and Arduino to create a Web server with a monitoring control function. Using the Wi-Fi library our device will be able to answer a HTTP request with the Wi-Fi shield. After opening a browser and navigating to the Wi-Fi shield's IP address, the Arduino will respond with just enough HTML for a browser to display the login webpage. The system should have access to an 802.11 b/g wireless network that connects to the Internet for this experiment. The system will need to change the network settings in the sketch to correspond to our particular networks SSID. The Wi-Fi shield uses pins 10, 11, 12, and 13 for the SPI connection to the HDG104 module. Digital pin 4 is used to control the slave select pin on the SD card. Figure 38 presents the transmitter side of our ZigBee network. Our router ZigBee modules transmit temperature and humidity data to coordinator side. Figure 39 shows the receiver side of our ZigBee network. Our coordinator ZigBee module receives temperature and humidity data that are displayed immediately on Arduino web server.

**Figure 40.**
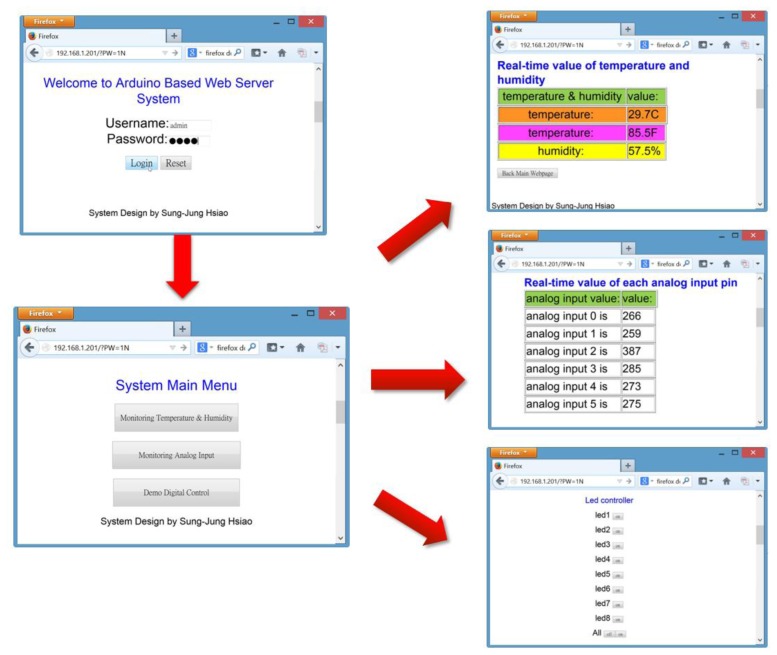
Arduino Based Web Server (ABWS) system.

Figure 40 is our Arduino Based Web Server (ABWS) system. The operator can view the login page “Welcome to Arduino Based Web Server System” heading. In the login page the administrator must enter the correct name and password to login to this monitoring system. The main page of the monitoring system has three options, including:

1. Monitoring Temperature & Humidity
2. Monitoring Analog Input
3. Digital Control

This is our early development status monitoring system with the basic structure formed. However, due to expansion the fact the functionality extension is limited, in this study we wrote an Android smartphone application.

### 8.2. Constructing App Programs to Android Smartphone

When this research utilizes the drawing functions to overcome the relevant program bottlenecks, this work uses the Android smartphone writing application. This system used a lot of graphics to compose our smartphone program to provide the user with a very user friendly interface. There is no way to write so many Web pages with graphics into the embedded chip. If pictures are written into the chip the system performance will degrade. The limited chip capacity prevents accessing a lot of pictures. This is why this study changed the program using Android writing for smartphones.

When this system executes the monitoring system program the system homepage will be presented. At this point this operator wants to login suing the username and password. If you enter the account correctly the system will turn on the main options page. System homepage Options include:

1. Monitoring factory zone situation
2. Loads running situation
3. Digital control

**Figure 41.**
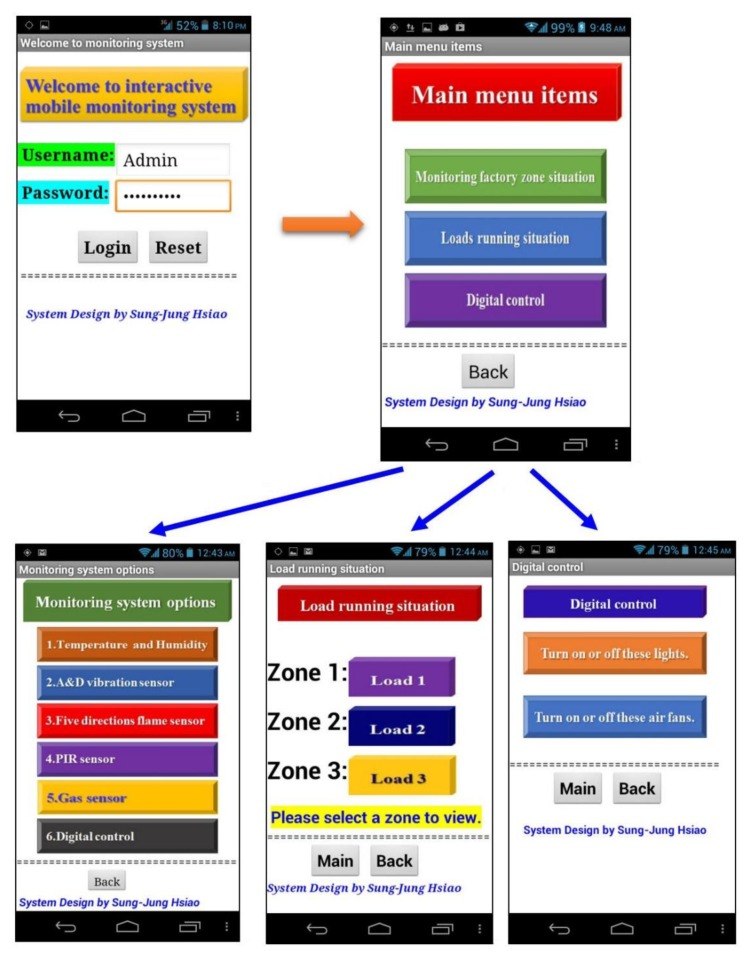
Our interactive monitoring system main menu structure.

Monitoring system options include a five-sensor monitoring project. The load running situation includes three monitoring areas. Digital control includes the lights and the fan on and off. Please refer to the detailed picture of Figure 41.

**Figure 42.**
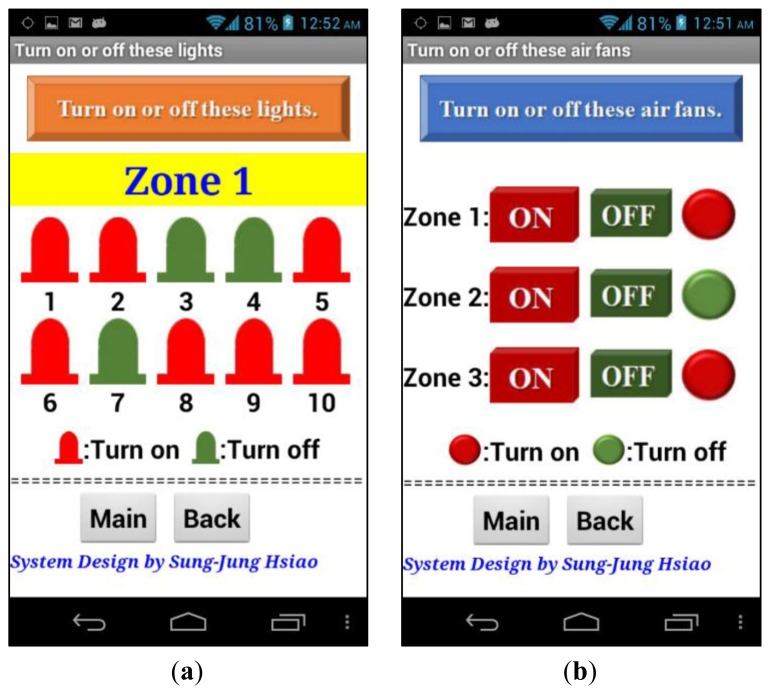
Interactive digital control to turn on or off these devices. (**a**) Turn on or off these lights; (**b**) Turn on or off these air fans.

The digital control is turned off and on using red and green, signifying closed or open. The LED button is used to turn on or off the factory area lights. Similarly, the red and green buttons are used to control the factory fan. The interactive digital control screen is shown in Figure 42.

This operator employs smart phone application writing that allows the operator to quickly and easily understand the status of each monitor.

## 9. Really Measured Load Simulation and Analysis

**Figure 43.**
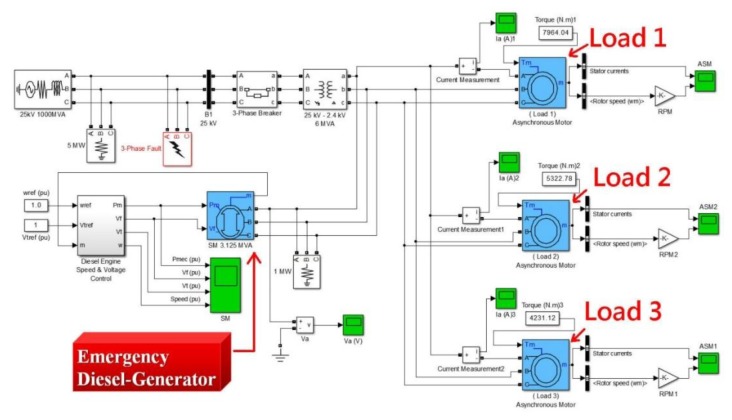
Factory load wiring diagram simulation.

There are three main motor loads in our factory. The entire power system includes diesel emergency generators to provide short term power when the system is interrupted. Figure 43 is a simplified diagram of the actual power system load graphic. Loads 1, 2 and 3 are located in the power system diagram on the right. The top left corner shows the power supply terminal. The emergency diesel generators are shown in the lower left. The load specifications for these three loads are quite different. This design allows us to use the Fast Fourier Transform to better distinguish between the time domain and frequency domain correctness, while allowing everyone more to easily understand the FFT analysis. In the actual analysis the loads will have no specification limits. These loads may be operated simultaneously or separately. Therefore, this work measured the total power supply to obtain the discrete voltage, current, power and frequency using the power measurement module. These data can be processed through the FFT to carry out load-side analysis. Table 2 presents the basic specifications for Loads 1, 2 and 3.

### 9.1. Actual Measured Data Using the Power Measurement Module

**Figure 44.**
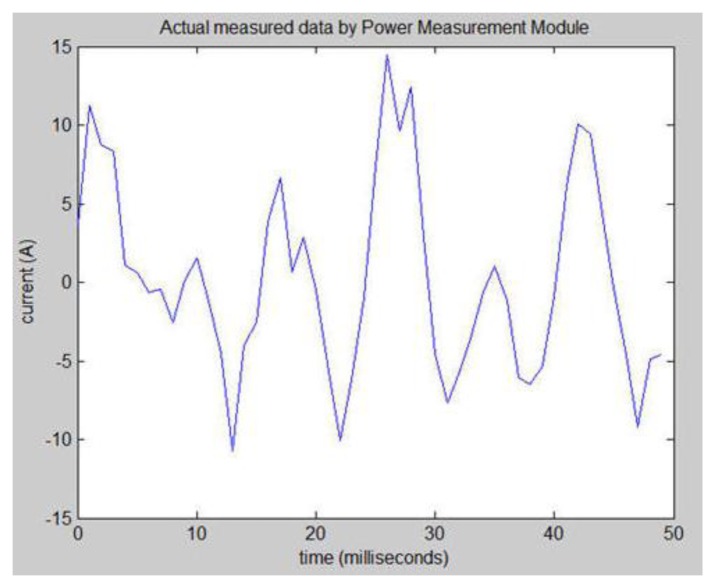
Actual measured data in millisecond units using the Power Measurement Module.

This section will describe the use of the Fast Fourier Transform (FFT) technique to analyze the factory load. The voltage, current, power, frequency, power factor and other discrete data are obtained first using the Power Measurement Module. The total power source load is measured. This system puts the total load current data utilization in millisecond sampling time units. This system total current data in milliseconds is used as the basic unit. This system actually measured discrete current data, in milliseconds, to map out the total current graph shown in Figure 44.

**Figure 45.**
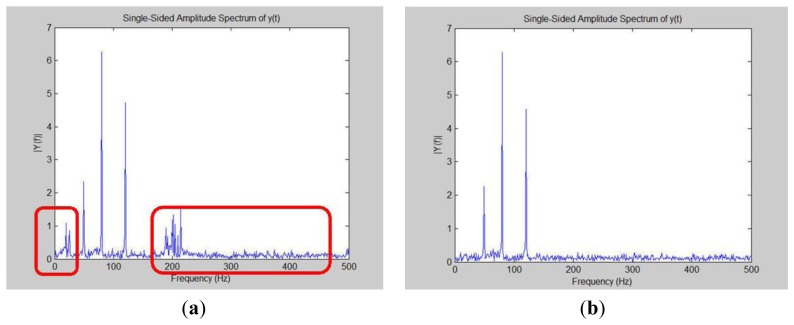
Time domain transformation into the frequency domain using FFT techniques. (**a**) Time domain into the frequency domain containing noises; (**b**) Time domain conversion into the frequency domain to remove these noises.

This research converts the discrete total current data from the time domain into the frequency domain using the FFT technique in Figure 45. This research analyzes Figure 45a to discover that the spectrum contains some noise. The noise is found in high and low places. High-frequency noise is especially obvious. This noise may interfere with our frequency domain analysis so this paper employs a filter. The specifications in Table 2 show the properties found is Loads 1, 2 and 3 in the operating frequency range between 30 Hz and 150 Hz. This system filters out high-frequency and low-frequency noise (refer to Figure 45b). This paper can clearly observe three different frequencies and amplitudes. These frequency bands are Loads 1, 2 and 3.

**Figure 46.**
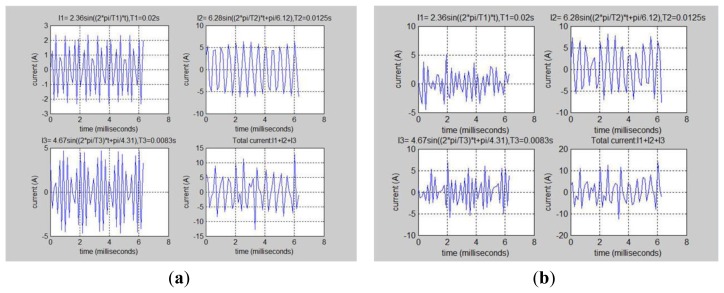
The ideal values are compared to the actual measured load current values. (**a**) Ideal value of load current; (**b**) Actual value of load current.

This research further analyzes Figure 45b to find the three load frequencies were 51.5 Hz, 83.2 Hz and 118.6 Hz. This analysis is very close to our actual measured values. The individually measured values were 50.2 Hz, 81.3 Hz and 120.1 Hz. Figure 46a is based on the running machine specification sheet drawn out Loads 1, 2 and 3 ideal individual loads current value charts. Figure 46b is the actual use of this power measurement module to measure the actual value.

### 9.2. Inverse Fast Fourier Transform (IFFT) Simulation

**Figure 47.**
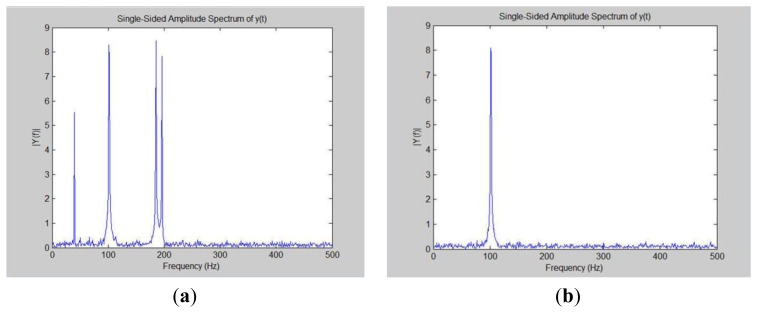
Interception and analysis of these frequencies. (**a**) Simulating the four load frequency domain; (**b**) Retaining only the needed load frequency.

Inverse Fast Fourier Transform (IFFT) is the inverse FFT. IFFT allows us to inverse frequency-domain data into the time domain data, revert to the previous module measurement data. Figure 47a sho–ws the spectrum of the four loads. The four load bands are very obvious. This research can clearly know the present total current frequency and in what range it is.

**Figure 48.**
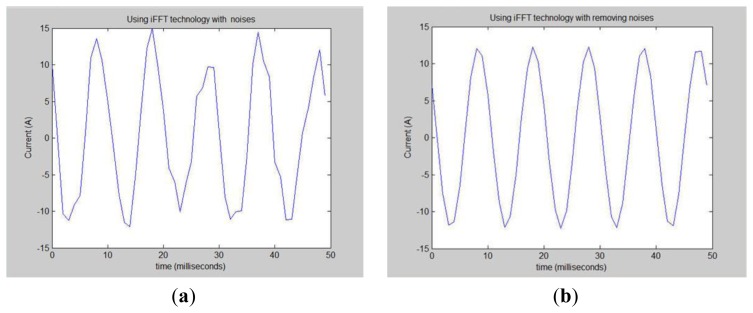
Using IFFT technology to restore the time domain.

Next this system wants to extract the frequency spectrum and then use the IFFT technique to restore the original load waveform. All of the frequency bands are transformed into the time domain using IFFT reduction techniques. Of course, restoring only a particular frequency is more accurate. Our experiments in Figure 47b only keep the frequency spectrum of about 100 Hz. When this research utilizes 100 Hz from a specific time to restore the original frequency domain, the waveform presented in Figure 48a.

Because Figure 48a contains some noise this study added some filters to filter out the noise. Figure 48b shows the noise removed in the time domain graph.

## 10. Conclusions

The plant operator can remotely log into this monitoring system via his smartphone. The factory area has a lot of very dangerous places that must be monitored using various sensors. This intelligent monitoring system is suitable for the management and control of a factory area. Managers using smart phones to instantly monitor a factory area are a very innovative approach. This work has obtained some new solutions for improved recognition, as follows:

- The Arduino based web-server approach uses the Wi-Fi and ZigBee network allowing managers to use a browser to connect to the Arduino-server with a smartphone. Managers can monitor the plant areas immediately after inputting their user-name and password.
- ZigBee protocol technology is used to send control signals to an integrated controller. This is an innovative method.
- The integrated controller uses an integrated protocol that includes ZigBee, RFID, Bluetooth, Infrared and Wi-Fi. A highly efficient module control is the specific contribution of this study.
- Fast Fourier Transform technology is employed to analyze the power source, allowing the monitor to immediately know the present electricity load situation. This research only supplies terminal measurement and analysis, so the system does not need to measure the load of each item. This is a new contribution using an innovative approach.

With further development the proposed monitoring system will be able to be widely applied to any dangerous industrial place. For example, chemical product manufacturing plants, cement product manufacturing plants or factories that employ highly polluting substances. Remote monitoring using smart phones to do the work is thus an excellent security application.

## Figures and Tables

**Figure 26.**
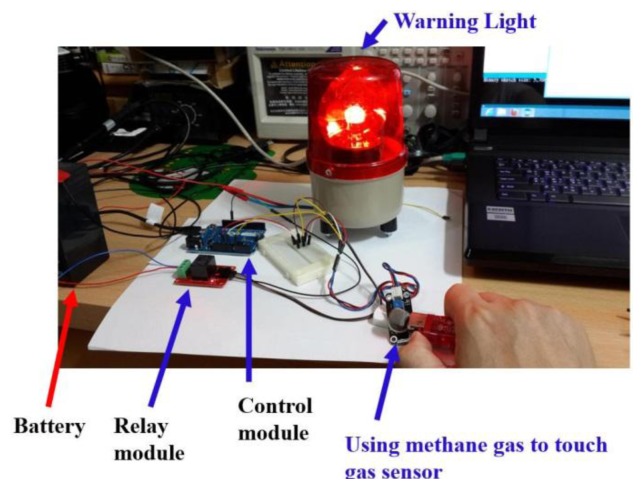
Experiments carried out with methane.

**Table 1.** The vibration sensor detects the voltage range corresponding to the actual situation.

**V_detection_ Range**	**V_detection_ < 80 mV**	**80mV < V_detection_ < 100 mV**	**V_detection_ > 100mV**
Situation	Safety	Caution	Danger

**Table 2.** Basic specifications for Loads 1, 2 and 3.

**Range**	**Load 1**	**Load 2**	**Load 3**
Voltage (V)	100 V–230 V	100 V–240 V	100 V–240 V
Current (A)	0–8 A	0–20 A	0–15 A
Frequency (Hz)	30–60 Hz	60–100 Hz	90–150 Hz
Rated Power (W)	1,840 W	4,800 W	3,600 W
Power Factor	0.42–0.56	0.73–0.86	0.62–0.66
Operating Temperature (°C)	32 °C–60 °C	40 °C–85 °C	36 °C–70 °C
Noise level	76 dB	90 dB	80 dB

**Figure A1.**
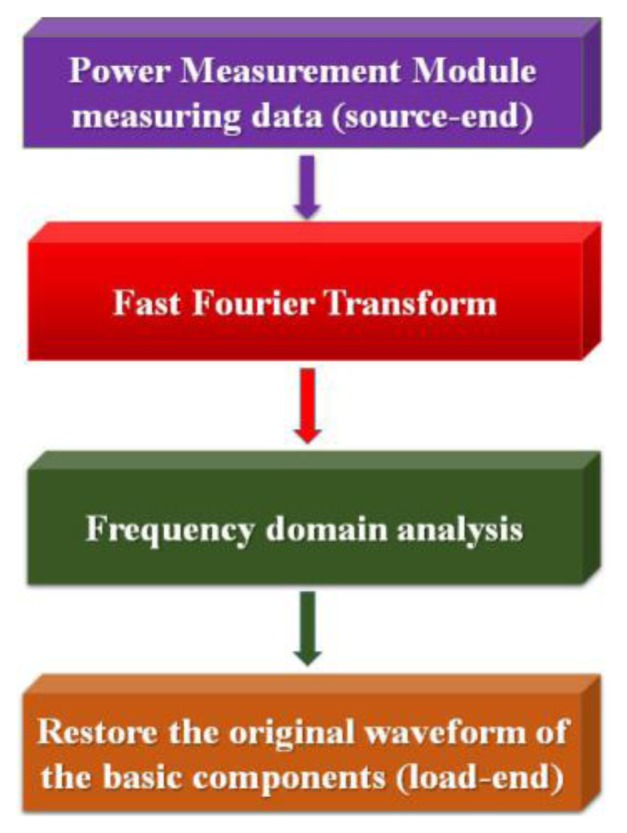
The proposed flow chart for power load analysis using the FFT approach.

**Figure A2.**
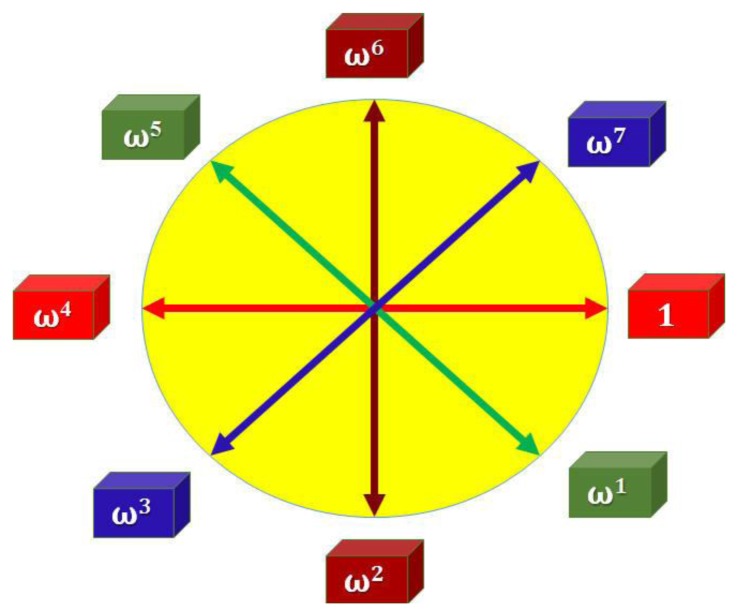
*ω* is symmetric, 8th roots of unity.

**Figure A3.**
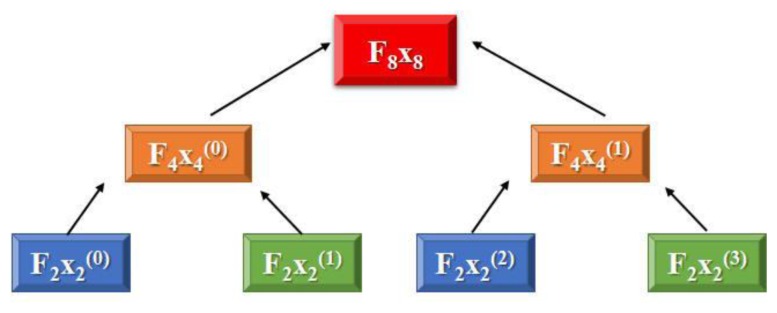
Fast Fourier transform of the divide-and-conquer process.

**Figure A4.**
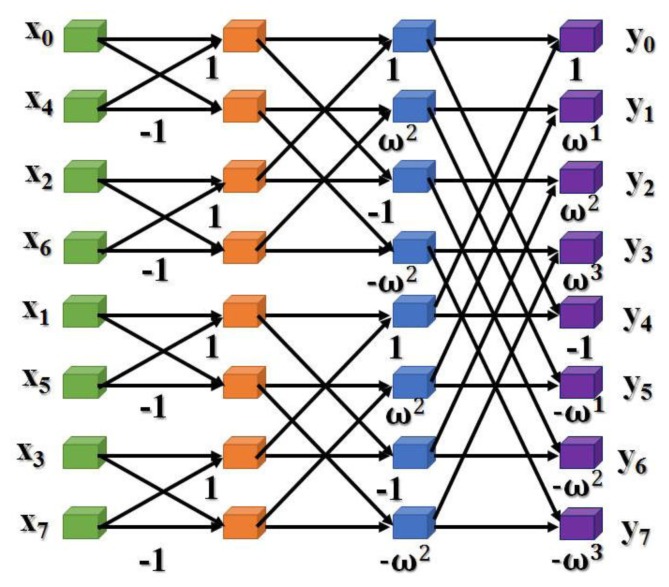
The FFT algorithm processes (when *n* = 8).

## Appendix

This section describes practical power measurement module application to analyze power source and load terminals. Figure A1 presents our proposed flow chart for power load analysis using the FFT approach [31,32]. First data stored in the Power Measurement Module is obtained. The the FFT unit calculates the frequency-amplitude analysis. Finally, the results are analyzed and then the unit reverts to the original basic waveform.

This system gets discrete data measured using Power Measurement Module, *x*_0_, *x*_1_… *x*_*n*-1_. The discrete Fourier transform is then calculated as:

(1)
$$\begin{array}{cc} y_{k}=\overset{n-1}{\underset{j=0}{\text{∑}}}x_{j}e^{-2\pi ikj/n}, & k=0,1,\ldots ,n-1 \end{array}$$

Order *ω* = *e*^−2^^*πi*/*n*^ = cos(2π/n) – isin(2π/n). Discrete Fourier transform can be expressed in a matrix form *y* = *Fx*, as follows:

(2)
$$\left[\begin{array}{c} y_{0}\\ y_{1}\\ y_{2}\\ \vdots \\ y_{n-1} \end{array}\right]=\left[\begin{array}{ccccc} 1 & 1 & 1 & \cdots & 1\\ 1 & w & w^{2} & \cdots & w^{n-1}\\ 1 & w^{2} & w^{4} & \cdots & w^{2(n-1)}\\ \vdots & \vdots & \vdots & \ddots & \vdots \\ 1 & w^{n-1} & w^{2(n-1)} & \cdots & w^{{\left(n-1\right)}^{2}} \end{array}\right]\;\left[\begin{array}{c} x_{0}\\ x_{1}\\ x_{2}\\ \vdots \\ x_{n-1} \end{array}\right]$$

where *n* × *n* order is called the Fourier matrix *F*. General matrix multiplication is used for discrete Fourier transform computation complexity of (*n*^2^). Cooley and Tukey presented a complexity of (*n* log_2_ *n*) algorithm, called the Fast Fourier Transform (FFT). The Fourier matrix F has a special nature. The block matrix techniques are derived from the Cooley-Tukey algorithm [33,34]. The Cooley-Tukey algorithm is the main idea for the sequence of length n divided into two discrete Fourier transforms of length *n*/2, sub-sequences of the discrete Fourier transform. To simplify the analysis, the following only considers the *n* = 2*^r^* situation. Order *F_n_* = [*f_pq_*] represents *n* × *n* Fourier matrix, the column line index is set to *p*,*q* ∈ {0, 1,…, *n*–1}, where *f_pq_* = *ω^pq^*, component units *ω* = *e*^−2^^*πi*/*n*^ has the following properties:

(1) complex numbers {1, *ω*, *ω*^2^,…, *ω^n^*^−1^} of the equation *z^n^* = 1 root, called *n*-th root of unity, the roots in the complex plane clockwise rotation on the unit circle and at the same pitch arrangement (*n* = 8, Figure A2 is an example). In addition, {1, *ω*^2^, *ω*^4^,…, *ω^n^*^−2^} is *z^n^*^/2^ = 1 for *n*/2 root of unity. Similarly, for *m* = 2*^k^*, *k* is a positive integer, {1, *ω^m^*, *ω^2m^*,…, *ω^n^*^−^*^m^*} is *z*^*n*/*m*^ = 1 to *n*/*m* root of unity, namely the establishment of a fast Fourier transform on top of this nature.
(2) *ω* is periodic. If *k* ≥ *n*, then *ω^k^* = *ω^k mod n^*, where *k mod n* represents the remainder by *k*/*n*. For example, if *n* = 8, then *ω*^8^ = 1, *ω*^9^ = *ω*, *ω*^10^ = *ω*^2^,….
(3) *ω* is symmetric, that is *ω^k^*^+^*^n^*^/2^ = −*ω^k^*, as *ω^p^* = cos(2πp/n) + isin(2πp/n), and:
  (3)
  $$\cos\left(2\pi \frac{k+\frac{n}{2}}{n}\right)=\cos\left(2\pi \frac{k}{n}+\pi \right)=\cos\left(2\pi \frac{k}{n}\right)$$
  (4)
  $$\sin\left(2\pi \frac{k+\frac{n}{2}}{n}\right)=\sin\left(2\pi \frac{k}{n}+\pi \right)=-\sin\left(2\pi \frac{k}{n}\right)$$

If *n* = 8, there is *ω^4^* = −1, *ω^5^* = −*ω*, *ω^6^* = −*ω^2^*, *ω^7^* = −*ω^3^*.

To facilitate the description this method derive the Cooley-Tukey algorithm using *n* = 8. Using properties (2) and (3), the 8 × 8 Fourier matrix can be simplified as:

(5)
$$\begin{array}{ll} F_{8} & =\left[\begin{array}{cccccccc} 1 & 1 & 1 & 1 & 1 & 1 & 1 & 1\\ 1 & \omega & \omega^{2} & \omega^{3} & \omega^{4} & \omega^{5} & \omega^{6} & \omega^{7}\\ 1 & \omega^{2} & \omega^{4} & \omega^{6} & 1 & \omega^{2} & \omega^{4} & \omega^{6}\\ 1 & \omega^{3} & \omega^{6} & \omega & \omega^{4} & \omega^{7} & \omega^{2} & \omega^{5}\\ 1 & \omega^{4} & 1 & \omega^{4} & 1 & \omega^{4} & 1 & \omega^{4}\\ 1 & \omega^{5} & \omega^{2} & \omega^{7} & \omega^{4} & \omega & \omega^{6} & \omega^{3}\\ 1 & \omega^{6} & \omega^{4} & \omega^{2} & 1 & \omega^{6} & \omega^{4} & \omega^{2}\\ 1 & \omega^{7} & \omega^{6} & \omega^{5} & \omega^{4} & \omega^{3} & \omega^{2} & \omega \end{array}\right]\\ & =[\begin{array}{cccccccc} 1 & 1 & 1 & 1 & 1 & 1 & 1 & 1\\ 1 & \omega & \omega^{2} & \omega^{3} & -1 & -\omega & -\omega^{2} & -\omega^{3}\\ 1 & \omega^{2} & -1 & -\omega^{2} & 1 & \omega^{2} & -1 & -\omega^{2}\\ 1 & \omega^{3} & -\omega^{2} & \omega & -1 & -\omega^{3} & \omega^{2} & -\omega \\ 1 & -1 & 1 & -1 & 1 & -1 & 1 & -1\\ 1 & -\omega & \omega^{2} & -\omega^{3} & -1 & \omega & -\omega^{2} & \omega^{3}\\ 1 & -\omega^{2} & -1 & \omega^{2} & 1 & -\omega^{2} & -1 & \omega^{2}\\ 1 & -\omega^{3} & -\omega^{2} & -\omega & -1 & -\omega^{3} & \omega^{2} & \omega \end{array}]\;, \end{array}$$

Re-using properties (1), the 4 × 4 Fourier matrix is:

(6)
$$F_{4}=\left[\begin{array}{cccc} 1 & 1 & 1 & 1\\ 1 & \omega^{2} & \omega^{4} & \omega^{6}\\ 1 & \omega^{4} & 1 & \omega^{4}\\ 1 & \omega^{6} & \omega^{4} & \omega^{2} \end{array}\right]=\left[\begin{array}{cccc} 1 & 1 & 1 & 1\\ 1 & \omega^{2} & -1 & -\omega^{2}\\ 1 & -1 & 1 & -1\\ 1 & -\omega^{2} & -1 & \omega^{2} \end{array}\right]$$

*F_8_* observation reveals even rows, namely 0,2,4,6 line *F_4_* constructed entirely, so this way will replace *F_8_* lines, even the top row, odd-numbered lines in the bottom:

(7)
$$P_{8}^{T}=\left[\begin{array}{cccccccc} {\text{e}}_{0} & {\text{e}}_{2} & {\text{e}}_{4} & {\text{e}}_{6} & {\text{e}}_{1} & {\text{e}}_{3} & {\text{e}}_{5} & {\text{e}}_{7} \end{array}\right]=\left[\begin{array}{cccccccc} 1 & 0 & 0 & 0 & 0 & 0 & 0 & 0\\ 0 & 0 & 0 & 0 & 1 & 0 & 0 & 0\\ 0 & 1 & 0 & 0 & 0 & 0 & 0 & 0\\ 0 & 0 & 0 & 0 & 0 & 1 & 0 & 0\\ 0 & 0 & 1 & 0 & 0 & 0 & 0 & 0\\ 0 & 0 & 0 & 0 & 0 & 0 & 1 & 0\\ 0 & 0 & 0 & 1 & 0 & 0 & 0 & 0\\ 0 & 0 & 0 & 0 & 0 & 0 & 0 & 1 \end{array}\right]$$

That was:

(8)
$$F_{8}P_{8}^{T}=\left[\begin{array}{cccccccc} 1 & 1 & 1 & 1 & 1 & 1 & 1 & 1\\ 1 & \omega^{2} & -1 & -\omega^{2} & \omega & \omega^{3} & -\omega & -\omega^{3}\\ 1 & -1 & 1 & -1 & \omega^{2} & -\omega^{2} & \omega^{2} & -\omega^{2}\\ 1 & -\omega^{2} & -1 & \omega^{2} & \omega^{3} & 1 & -\omega^{3} & -\omega \\ 1 & 1 & 1 & 1 & -1 & -1 & -1 & -1\\ 1 & \omega^{2} & -1 & -\omega^{2} & -\omega & -\omega^{3} & \omega & \omega^{3}\\ 1 & -1 & 1 & -1 & -\omega^{2} & \omega^{2} & -\omega^{2} & \omega^{2}\\ 1 & -\omega^{2} & -1 & \omega^{2} & -\omega^{3} & -\omega & \omega^{3} & \omega \end{array}\right]=\left[\begin{array}{cc} F_{4} & D_{4}F_{4}\\ F_{4} & -D_{4}F_{4} \end{array}\right]$$

In the above formula, *D*_4_ = diag(1, *ω*, *ω*^2^, *ω*^3^), carefully, the {1, *ω*, *ω*^2^, *ω*^3^} is the first four roots of *z*^8^ = 1, generally summarized as follows:

Similarly make *ω* = *e*^−2*πi*/*n*^, for *m* = 2*^k^* ≤ *n*, *k* is a positive integer, as $P_{m}^{-1}=P_{m}^{T}$, the *m* × *m* Fourier matrix *F_m_* partakers block matrix expression:

(9)
$$F_{m}=\left[\begin{array}{cc} F_{\frac{m}{2}} & D_{\frac{m}{2}}F_{\frac{m}{2}}\\ F_{\frac{m}{2}} & -D_{\frac{m}{2}}F_{\frac{m}{2}} \end{array}\right]\;P_{m}$$

where *F*_*m*/*2*_ is *m*/*2* × *m*/*2* Fourier matrix, *D*_*m*/*2*_ = diag(1, *ω*^*n*/*m*^, *ω*^2^^*n*/*m*^,…, *ω*^*n*/*2*–*n*/*m*^), main diagonal of *z^m^* = 1 the first *m*/*2* roots, and $P_{m}^{T}=\left[\begin{array}{l} e_{0}e_{2}\ldots e_{m-2}e_{1}e_{3}\ldots e_{m-1} \end{array}\right]$.

The *n* = 8 as an example below to show the fast Fourier transform calculus process. Subject to transform a given vector *x*_8_ its replacement results are as follows:

(10)
$$\begin{array}{ccc} {\text{x}}_{8}=\left[\begin{array}{c} x_{0}\\ x_{1}\\ x_{2}\\ x_{3}\\ x_{4}\\ x_{5}\\ x_{6}\\ x_{7} \end{array}\right], & {\text{P}}_{8}{\text{x}}_{8}=\left[\begin{array}{c} x_{0}\\ x_{2}\\ x_{4}\\ x_{6}\\ x_{1}\\ x_{3}\\ x_{5}\\ x_{7} \end{array}\right]= & \left[\begin{array}{c} {\text{x}}_{4}^{\left(0\right)}\\ --\\ {\text{x}}_{4}^{\left(1\right)} \end{array}\right] \end{array}$$

where $x_{4}^{\left(0\right)}$ and $x_{4}^{\left(1\right)}$respectively, by *x*_8_ the even and odd elements composition. Using block matrix multiplication, this result can obtain:

(11)
$$\begin{array}{ccc} F_{8}{\text{x}}_{8}=\left[\begin{array}{cc} F_{4} & D_{4}F_{4}\\ F_{4} & -D_{4}F_{4} \end{array}\right] & P_{8}{\text{x}}_{8}=\left[\begin{array}{cc} F_{4} & D_{4}F_{4}\\ F_{4} & -D_{4}F_{4} \end{array}\right]\left[\begin{array}{c} {\text{x}}_{4}^{\left(0\right)}\\ {\text{x}}_{4}^{\left(1\right)} \end{array}\right]=\left[\begin{array}{c} F_{4}{\text{x}}_{4}^{\left(0\right)}+D_{4}F_{4}{\text{x}}_{4}^{\left(1\right)}\\ F_{4}{\text{x}}_{4}^{\left(0\right)}-D_{4}F_{4}{\text{x}}_{4}^{\left(1\right)} \end{array}\right] & \left(11\right) \end{array}$$

The above Equation clearly shows the fast Fourier transform of divide-and-conquer process: The eight factorial method *F*_8_*x*_8_ replaced by two 4-factorial methods $F_{4}x_{4}^{\left(0\right)}$ and $F_{4}x_{4}^{\left(1\right)}$ Using a recursive strategy continue to break down, so:

(12)
$$\begin{array}{cc} P_{4}{\text{x}}_{4}^{\left(0\right)}=\left[\begin{array}{c} x_{0}\\ x_{4}\\ x_{2}\\ x_{6} \end{array}\right]=\left[\begin{array}{c} x_{2}^{\left(0\right)}\\ --\\ x_{2}^{\left(1\right)} \end{array}\right], & P_{4}{\text{x}}_{4}^{\left(1\right)}=\left[\begin{array}{c} x_{1}\\ x_{5}\\ x_{3}\\ x_{7} \end{array}\right]=\left[\begin{array}{c} x_{2}^{\left(2\right)}\\ --\\ x_{2}^{\left(3\right)} \end{array}\right] \end{array}$$

where:

(13)
$$\begin{array}{cccc} F_{4}{\text{x}}_{4}^{\left(0\right)}=\left[\begin{array}{cc} F_{2} & D_{2}F_{2}\\ F_{2} & -D_{2}F_{2} \end{array}\right] & P_{4}{\text{x}}_{4}^{\left(0\right)}=\left[\begin{array}{cc} F_{2} & D_{2}F_{2}\\ F_{2} & -D_{2}F_{2} \end{array}\right]\;\left[\begin{array}{c} {\text{x}}_{2}^{\left(0\right)}\\ {\text{x}}_{2}^{\left(1\right)} \end{array}\right] & = & \left[\begin{array}{c} F_{2}{\text{x}}_{2}^{\left(0\right)}+D_{2}F_{2}{\text{x}}_{2}^{\left(1\right)}\\ F_{2}{\text{x}}_{2}^{\left(0\right)}-D_{2}F_{2}{\text{x}}_{2}^{\left(1\right)} \end{array}\right] \end{array}$$

(14)
$$\begin{array}{cccc} F_{4}{\text{x}}_{4}^{\left(1\right)}=\left[\begin{array}{cc} F_{2} & D_{2}F_{2}\\ F_{2} & -D_{2}F_{2} \end{array}\right] & P_{4}{\text{x}}_{4}^{\left(1\right)}=\left[\begin{array}{cc} F_{2} & D_{2}F_{2}\\ F_{2} & -D_{2}F_{2} \end{array}\right]\;\left[\begin{array}{c} {\text{x}}_{2}^{\left(2\right)}\\ {\text{x}}_{2}^{\left(3\right)} \end{array}\right] & = & \left[\begin{array}{c} F_{2}{\text{x}}_{2}^{\left(2\right)}+D_{2}F_{2}{\text{x}}_{2}^{\left(3\right)}\\ F_{2}{\text{x}}_{2}^{\left(2\right)}-D_{2}F_{2}{\text{x}}_{2}^{\left(3\right)} \end{array}\right] \end{array}$$

and $D_{2}=\left[\begin{array}{cc} 1 & 0\\ 0 & w^{2} \end{array}\right]$, because $F_{2}=\left[\begin{array}{cc} 1 & 1\\ 1 & w^{4} \end{array}\right]=\left[\begin{array}{cc} 1 & 1\\ 1 & -1 \end{array}\right]$, Very easy to be considered, $F_{2}x_{2}^{\left(j\right)}$, *j* = 0,1,2,3. Based on the above discussion, the fast Fourier transform divide and conquer operations can be expressed as the following tree as Figure A3.

The following Figure A4 shows the *n* = 8 Cooley-Tukey algorithm processes. If *n* = 2*^r^*, the Fast Fourier Transform is decomposed into *r* = log_2_*n* layers, each with *n*/2 multiplication (not marked in the figure representing a linear multiplier “multiplied by 1” and therefore can be disregarded), so the total multiplications as $\frac{n}{2}=\log_{2}n$.

Procedure FFT is presented here in pseudo-code, for a generic field *F* in which it is possible to define *ω*, a primitive *n*-th root of unity.

Preconditions:

(1) A is a Vector of length *n*
(2) *n* is a power of 2
(3) *ω* is a primitive *n*-th root of unity.

The Vector A represents the polynomial a(z) = A[1] + A[2] × z + … + A[n] × z^(n − 1)^.

The value returned is a Vector of the values [a(1), a(*ω*), a(*ω^2^*), …, a (*ω*^(^*^n^*^− 1)^)] computed via a recursive FFT algorithm.

Procedure FFT (A, n, ω)
If n = 1 then:
return A
Else:
Aeven ←Vector([A[1], A[3], …, A[n–1]])
Aodd ← Vector([A[2], A[4], …, A[n]])
Veven ← FFT(Aeven, n/2, ω̂2)
Vodd ← FFT(Aodd, n/2, ω̂2)
V ← Vector(n) // Define a Vector of length n
For i from 1 to n/2 do
V[i] ← Veven[i] + ω̂(i–1)*Vodd[i]
V[n/2+ i] ←Veven[i] – ω̂(i–1)*Vodd[i]
End do
return V
End If
End procedure
